# Characterisation of Type II DNA Methyltransferases of *Metamycoplasma hominis*

**DOI:** 10.3390/microorganisms11061591

**Published:** 2023-06-15

**Authors:** Lars Vogelgsang, Azlan Nisar, Sebastian Alexander Scharf, Anna Rommerskirchen, Dana Belick, Alexander Dilthey, Birgit Henrich

**Affiliations:** Institute of Medical Microbiology and Hospital Hygiene, Medical Faculty of the Heinrich-Heine-University Duesseldorf, Universitätsstraße 1, 40225 Duesseldorf, Germany; lars.vogelgsang@hhu.de (L.V.); azlan@uni-duesseldorf.de (A.N.); sebastianalexander.scharf@med.uni-duesseldorf.de (S.A.S.); anna.rommerskirchen@med.uni-duesseldorf.de (A.R.); dana.belick@hhu.de (D.B.); alexander.dilthey@med.uni-duesseldorf.de (A.D.)

**Keywords:** *dam*, *dcm*, DNA MTase, *Metamycoplasma hominis*, RM system, orphan, Nanopore, Tombo

## Abstract

Bacterial virulence, persistence and defence are affected by epigenetic modifications, including DNA methylation. Solitary DNA methyltransferases modulate a variety of cellular processes and influence bacterial virulence; as part of a restriction-modification (RM) system, they act as a primitive immune system in methylating the own DNA, while unmethylated foreign DNA is restricted. We identified a large family of type II DNA methyltransferases in *Metamycoplasma hominis*, comprising six solitary methyltransferases and four RM systems. Motif-specific 5mC and 6mA methylations were identified with a tailored Tombo analysis on Nanopore reads. Selected motifs with methylation scores >0.5 fit with the gene presence of DAM1 and DAM2, DCM2, DCM3, and DCM6, but not for DCM1, whose activity was strain-dependent. The activity of DCM1 for C^m^CWGG and of both DAM1 and DAM2 for G^m^ATC was proven in methylation-sensitive restriction and finally for recombinant rDCM1 and rDAM2 against a *dam*-, *dcm*-negative background. A hitherto unknown *dcm*8/*dam*3 gene fusion containing a (TA) repeat region of varying length was characterized within a single strain, suggesting the expression of DCM8/DAM3 phase variants. The combination of genetic, bioinformatics, and enzymatic approaches enabled the detection of a huge family of type II DNA MTases in *M. hominis*, whose involvement in virulence and defence can now be characterized in future work.

## 1. Introduction

*Metamycoplasma hominis* is a facultative pathogen of the human urogenital tract, controversially discussed to be associated with bacterial vaginosis, pelvic inflammatory disease, preterm birth [1], and disseminated infections, such as septic arthritis, (neonatal) meningitis, or abscesses [2,3]. *M. hominis* belongs to the class of the cell wall-less mollicutes that have evolved from gram-positive species by reductive evolution, initiated by a parasitic life on the mucosal surfaces of the host with the result of a minimized genome and limited metabolic activity [4]. Against this rather unexpected background, mycoplasmas possess a large repertoire of mobile genetic elements (MGEs), defence islands, and genomic hotspots, which account for their phenotypic intra-species heterogeneity [5]. DNA methyltransferases (MTases) were found to be encoded by genes of hotspots and defence islands, either solitary or as part of a restriction-modification (RM) system [6]. RM systems were more often detected in small MGEs that were sometimes affected by degradation, resulting in the expression of residual RM genes, whereas solitary MTase genes are transferred by large MGEs [7].

Methylation is the main epigenetic modification of bacterial DNA [8], and the repertoire of DNA MTases represents a characteristic signature of the respective strain, associated with vitality, defence, and virulence [9]. DNA MTases transfer a methyl group from S-adenosine-L-methionine (SAM or AdoMet) to the N6 position of adenine (6mA MTases) or the N4 or C5 position of cytosine (4mC or 5mC MTases) of a specific recognition sequence [10]. The exocyclic N-targeting, N-6-adenine, and N-4-cytosine MTases contain nine conserved domains [11] and are subdivided into six groups (α, β, γ, ξ, δ, and ε) due to their SAM binding site (motifs X, I–III), catalytic site (motifs IV–VIII), and target recognition site (TRD) [12], whereas C-5 MTases contain 10 motifs (I–X) in a common two-domain architecture ([I–VIII]—TRD-[IX–X] [13,14,15]), of which six motifs (I, IV, VI, VIII, IX, and X) are higher conserved [15,16,17].

DNA methylations by solitary MTases are known to affect gene regulation [9,18] or modulate various cellular processes, such as DNA repair, cell cycle control, or mismatch repair [19,20,21]. RM systems primarily serve as a rudimentary bacterial immune system to defend against foreign DNA [22,23]. Protecting their own DNA with a specific methylation of a sequence motif (MTase activity), the foreign DNA (e.g., derived from bacteriophages [24]) will be restricted at the respective non-methylated DNA-motif (REase activity). Over 3000 different RM systems have been identified in prokaryotes to date [25], of which the largest class comprises type II RM systems. MTase and REase of type II RM systems are organized in the same operon, but they act independently on short (usually 4–8 bp) palindromic recognition sequences.

In mycoplasmas, DNA methylation and associated RM systems have been poorly studied. A large-scale study by Dordet-Frisoni et al. examined the effects of *M. agalactiae* RM systems on genome plasticity and pan-epigenomic dynamics [26]. Samashko et al. characterized the type I RM system dependent downregulation of gene expression in *M. gallisepticum* to depend on 6mA modification of the promoter region in the -10-box [27]; Chernov et al. demonstrated for *M. hyorhinis*, a host cell-invasive mycoplasma species, that special G^m^ATC- and ^m^CG-specific MTases can selectively and efficiently methylate the host genome, thus altering the epigenetic landscape in human cells [28]. A comprehensive characterisation of the methylome of *M. genitalium* and *M. pneumoniae* was done in using SMRT (Single-Molecule-Real-Time) sequencing and led to the identification of a formerly unknown bacterial 6mA methylation motif (CT**^m^A**T) [29]. In *M. hominis*, an MTase gene was detected in type strain ATCC23114 by comparative genomics and later characterized as conserved part on ICEHo-I, an integrative and conjugative element [13,30,31]. Apart from this postulated C5-cytosine DNA MTase (named DCM1 in this study), no further 5mC, 4mC, or 6mA type II DNA MTases had been characterised in *M. hominis* so far. The aim of this study was to fill this knowledge gap by characterizing novel MTase homologs.

## 2. Material and Methods

### 2.1. Bacterial Culturing and Nucleic Acid Preparations

*M. hominis* strains, which were isolated from human specimens, were cultivated in arginine medium as described in detail previously [32]. *Escherichia coli* clones were cultivated overnight in LB medium supplemented with 100 mg/L ampicillin and 25 mg/L chloramphenicol.

For qPCR analysis, cell sediment of 1 mL culture was suspended in 200 µL proteinase K solution (66 µg/mL Proteinase K (Roche, Mannheim, Germany)/3.3 mM Tris/HCl, pH 8.0) and incubated for 1 h at 56 °C. After heat inactivation of the proteinase K for 30 min at 95 °C, insoluble material of the cell lysate was sedimented at 13,000× *g* for 10 min. The DNA containing supernatant was stored at −20 °C until use in PCR.

For restriction analysis, genomic DNA of bacterial strains was isolated by the use of the DNeasy Blood and Tissue kit (Qiagen, Hilden, Germany) following the tissue protocol with minor modifications for mycoplasma DNA as published [33].

For RT-PCR analysis, RNA was prepared with the RNeasy Kit (Qiagen GmbH, Hilden, Germany) and cDNA synthesised as published [34].

Concentration of genomic DNA and RNA was measured by Invitrogen Qubit 4 Fluorometer Qubit (ThermoFisher Scientific, Waltham, MA, USA). Quality of DNA and RNA was verified by NanoDrop 1000 Spectrophotometer (ThermoFisher Scientific, Waltham, MA, USA).

### 2.2. qPCR

Oligonucleotides used in qPCRs were designed using Probefinder (Roche Applied Science, Penzberg, Germany) (https://qpcr.probefinder.com, accessed on 17 February 2022) or PrimerSelect of DNASTAR (Madison, WI, USA). Primers are listed in Table 1. 

qPCR assays were carried out in a total volume of 25 μL consisting of 1 × MesaGreen MasterMix (incl. reaction buffer, 4 mM MgCl_2_, dNTPs (N = A, C, G, T, U), Meteor *Taq* polymerase, SYBRGreen I, stabilizers, and fluorescein), 300 nM of each primer, and 2.5 μL of genomic DNA or cDNA solution, which was derived from 100 ng RNA [34]. Thermal cycling conditions were as follows: 1 cycle at 50 °C for 10 min, 1 cycle at 95 °C for 5 min, and 35 cycles at 95 °C for 15 s and 60 °C for 1 min. The product was then heated from 6 °C to 95 °C with an increment of 0.5 °C/15 s, and the plate read for melt curve analysis to check the identity of the amplicon. Cycling, fluorescent data collection, and analysis were carried out on a CFX-Cycler of BioRad Laboratories (Munich, Germany) according to the manufacturer’s instructions. Ct values were interpreted relative to the chromosomal *M. hominis*-specific *hit*A gene (genomic DNA) or the mean of three (housekeeping) genes *gap*, *lgt*, and mho_0150 (cDNA), which were shown to be differentially unregulated as formerly published [31,34]. Ct values >30 were interpreted as negative.

### 2.3. Methylation Sensitive Restriction (MSR) Analysis

Genomic DNA (0.75 µg) was digested with 3 U restriction enzyme in 15 µL for 1 h at 37 °C followed by heat inactivation of the enzyme at 80 °C for 20 min. Methylation specificities are listed in Table 2. Restricted DNA was analysed on 1% agarose gels.

### 2.4. Heterologous Expression of rDCM1 and rDAM2

Open reading frames of *dcm*1 (isolate FBG) and *dam*2 (isolate 8958) were codon optimized for best expression in *E. coli* and cloned into *Bam*HI-*Hin*DIII sites of pcDNA3.1(-)-myc-HisA (GenScript Biotech B.V., Leiden, The Netherlands). Plasmids were propagated into *dam*/*dcm*-deficient *E. coli* (New England Biolabs GmbH, Frankfurt am Main, Germany) and cultivated in LB medium supplemented with 100 mg/L ampicillin and 25 mg/L chloramphenicol. Heterologous expression of His_6_-tagged proteins was documented in His_4_-immunostaining as formerly published [35].

### 2.5. Tailored Tombo-Analysis

The present study was based on a re-analysis of Nanopore sequencing data generated for an earlier study [31]; full details on sample preparation and sequencing data generation are given in Henrich et al. (2020) [31]. Briefly, *M. hominis* isolates were sequenced on a MinION MK1B device (Oxford Nanopore Technologies, Oxford, UK); sequencing libraries from quality-controlled genomic DNA were prepared according to the manufacturer’s instructions and sequenced using the barcode ligation-based protocol. The methylation status for each cytosine and adenine in the analyzed mycoplasma isolates was determined with Tombo 1.5 (https://github.com/Nanoporetech/Tombo) [36], and the results were processed in R 4.2.1 [37]. The normalized signal was base-called by Tombo, and a methylation score was calculated for the specified bases. Modified base detection for 5mC and 6mA was calculated with the alternative 5mC and 6mA models, which are implemented in Tombo (parameter --alternate-bases 6mA 5mC). Output in bigwig format was specified via the parameter -–file-types coverage dampened_fraction. Motif-specific methylation scores were calculated by determining motif positions relative to the reference genome in R version 4.2.1 [36] and by extracting the methylation scores of the corresponding motif-contained A and C bases. A or C reference genome positions for which no methylation score was produced by Tombo were ignored; the effect of this was confirmed to be negligible (<1–2% of relevant positions per isolate). Visualization of methylation scores was carried out with the boxplot function in R.

### 2.6. Bioinformatic Analyses

Multiple sequence alignments were calculated using Genious Pro vers. 5.5.8 (Dotmatics, Boston, MA, USA) or MegAlign version 5.08 of the Lasergene software package (DNAStar, Madison, WI, USA). Genome alignments illustrating gene gain, loss, and rearrangement were performed with Genious. BLASTP 2.14.0 (https://blast.ncbi.nlm.nih.gov/Blast.cgi?PAGE=Proteins, accessed on 25 June 2022) and Phyre2 ver. 2.0 web portal (http://www.sbg.bio.ic.ac.uk/phyre2/html/page.cgi?id=index) [38] were used for protein prediction and analysis. REBASE ver. 306 (http://rebase.neb.com/rebase/rebase.html, accessed on 5 June 2022) was used for the prediction of restriction-modification systems and related proteins/MTases.

### 2.7. Statistical Analyses

Statistical analysis of the prevalence of (active) MTases and MGEs was carried out in Python version 3.8.16 using Jupyter Notebook version 6.4.12 (Project Jupyter, https://jupyter.org/). For analyses, the pandas version 1.4.3 and SciPy version 1.10.0 modules were primarily employed [37]. For Mann–Whitney U and Kruskal–Wallis tests, *p*-values were calculated using SciPy. No multiple testing corrections were used. For correlation analysis, correlation coefficients and *p*-values were calculated using Pearson’s R test. Visualization was performed using seaborn package version 0.11.2 [38].

## 3. Results

### 3.1. Detection of Type II MTases

**Figure 1:**
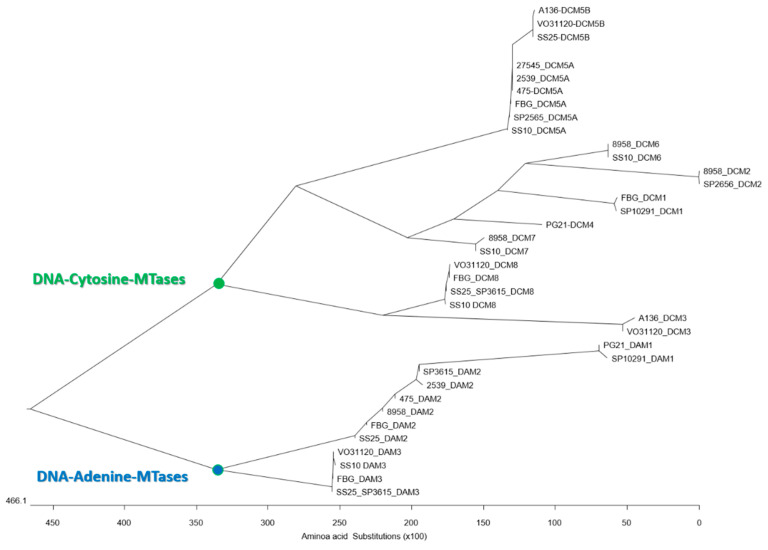
Phylogenetic tree of type II DNA MTases presents two main branches of DAM and DCM homologs in *M. hominis.* MTase sequences of the *M. hominis* strains tested, whose accession numbers are listed in Supplementary Table S1, were used in ClustalW-based Multiple Sequence Alignment using software program MegAlign 5.08 with default setting for phylogenetic tree construction.

In a BLASTP analysis, two *M. hominis* proteins of different lengths were detected as the highest homolog to the *E. coli* DAM protein sequence (283 AA; acc-no. QNJ69579.1): a 442 AA protein with 64% coverage and 32% AA identity in type strain PG21 (WP_012855752.1; named DAM1) and a 282 AA protein with 85% coverage and 34% identity in strain 8958 (WP_052498742.1; named DAM2). In a REBASE analysis, *dcm*1, *dam*1, and *dam*2 genes were detected in 9, 2, and 15 (8/15 with truncation) of 23 *M. hominis* genomes [31], respectively, and further gene loci identified encoding type II DNA MTases. A multiple protein sequence alignment was used for the construction of a phylogenetic tree (Figure 1). DCM1 and DAM1/DAM2 clustered in two separate branches, suggesting that members of one branch act as DNA-cytosine and the other as DNA-adenine MTases.

The DNA MTase protein sequences of *M. hominis* strains were aligned with ClustalW for the phylogenetic tree construction. Based on the phylogenetic branching, three different DNA-adenine MTases (DAM1 to DAM3) and eight DNA-cytosine MTases (DCM1 to DCM8) were postulated in the *M. hominis* strains tested.

**Figure 2:**
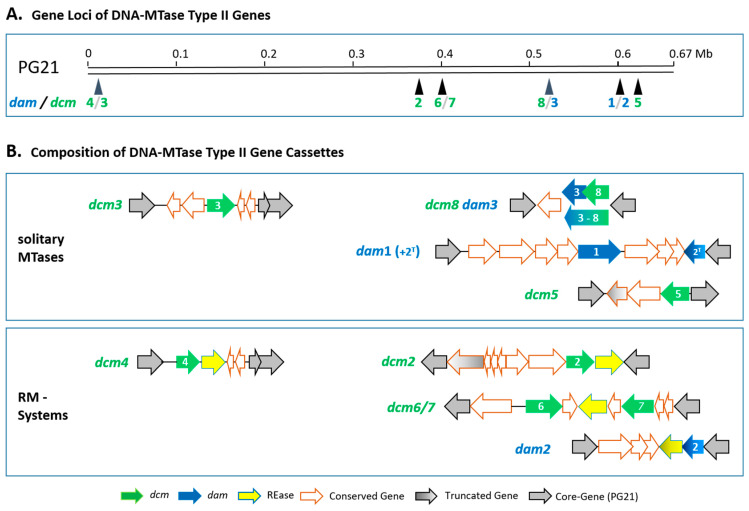
Type II DNA MTase gene loci and cassettes. (**A**) Position of the DNA MTase gene loci with respect to the genome sequence of ATCC23114 (type strain PG21); *dam* loci (1–3) in blue, *dcm* loci (1–8) in green. (**B**) Composition of the MTase gene loci. Genes are represented by arrows and coloured as follows: chromosomal core genes (accord. to PG21) in grey, conserved genes in the MTase cassettes (red), *dam* homologs in blue, *dcm* homologs in green, restriction endonuclease (REase) genes in yellow with blue frame; genes, in some isolated affected by truncation, are shaded.

The position of the DNA MTase genes was highly conserved; *dcm*1 was always present on the mobile genetic element ICEHo-I [30,31], although the integration site of the ICEHo-I element in the genomes was random. The localisation of the other MTase genes is schematically shown in Figure 2A with respect to type strain PG21 (ATCC 23114).

In addition to *dcm*1, five further solitary (orphan) MTase genes (*dam*1, *dam*3, *dcm*3, *dcm*5, and *dcm*8) and five MTase genes of RM systems (*dam*2, *dcm*2, *dcm*4, *dcm*6+*dcm*7) were predicted with the REBASE analysis (Figure 2B). The architecture of some MTase gene loci was strongly conserved (*dam*1, *dcm*1, *dcm*3, *dcm4*, *dcm*6+*dcm*7, and *dcm8*/*dam*3), whereas others were affected by strain-specific deletions and/or insertions of gene cassettes (*dam*2, *dcm*2, and *dcm*5; see Supplementary Figure S1). Four gene loci were targeted by two different MTase genes: *dcm*3 and *dcm*4; *dcm*6 and *dcm*7; *dcm8* and *dam*3; and *dam*1 and *dam*2, respectively. The orphan MTase *dcm*3 and the *dcm*4 RM system both targeted the same genomic region corresponding to the PG21 region between genes MHO_0350 and MHO_0380. As they did not share sequence homologies to one another, a common ancestor or *dcm*4/REase-fusion to form *dcm*3 was excluded. For *dam*3 and *dcm*8, a rare fusion event was assumed in isolate SS10. As the fusion did not lead to a frameshift, the activity of both MTase parts was assumed. *Dcm*6 and *dcm*7 always occurred as RM systems in a conserved gene cassette, which was located between the PG21 homolog region between genes MHO_3280 and MHO_3300. The REase and *dcm*7 gene were positioned on the same coding strand, and *dcm*6 was on the opposite. A similar architecture was found for the *dam*1 and the *dam*2 RM system, both located in a genome region between MHO_4730 and MHO_4840, with *dam*1 on one strand and the *dam*2 RM system on the other. This gene locus was more diverse, but the same set of additional genes (MHO_4790-MHO_4810) was present in some strains. In contrast to *dcm*6/7, the *dam*1 and full-length *dam*2 genes did not occur simultaneously. If the *dam*2/REase-RM system was unscathed (in strains 8958, 475, SS25, SP3615) or *dam*2 truncated at the 5′-end (strains FBG or 2539), *dam*1 was never found. In strains with *dam*1 (PG21, SP10291), only fragments of the *dam*2 gene (pseudogenes) were present, and the REase gene was always absent (see Supplementary Figure S1).

### 3.2. Prevalence of Type II MTases

**Figure 3:**
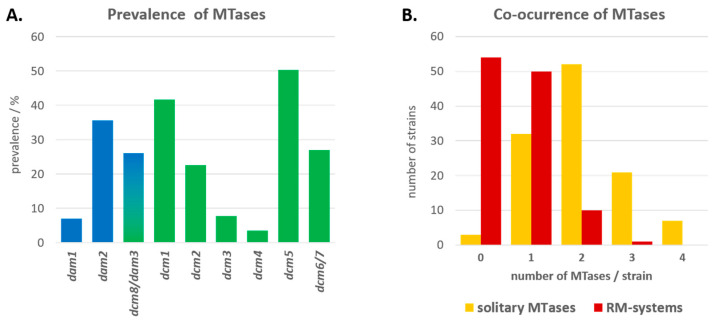
Prevalence and co-occurrence of type II DNA MTases in *M. hominis*. (**A**) Bargraphs of the different *dam* (blue) and *dcm* (green) gene prevalence (%) calculated on presence in the 115 *M. hominis* strains tested (Kruskal-Wallis *p* = 2.33 × 10^−54^). (**B**) Bargraphs of the number of *M. hominis* strains with zero to four different solitary MTases (orange) or MTases of RM systems (red). The combination of *dam* and *dcm* genes in view on solitary- and RM-MTases is listed in Table S3 for all strains (Mann–Whitney U *p* = 0.556).

Using a real time PCR (qPCR) screening approach, 115 isolates from the *M. hominis* strain collection of our institute were tested for the presence of *dam* and *dcm* homologues and related restriction endonuclease genes. To verify the accuracy of the qPCR screen, the already long-read sequenced genomes served as a control (Supplementary Table S2). The qPCR-based results were in perfect agreement for the sequenced strains. An amount of 55.7% (64/115) of the isolates carried *dam* homolog genes, and 93.9% (108/115) carried *dcm* homolog genes. Only 3/115 isolates were *dam*- and *dcm*-free. The prevalence of *dam*1 and *dam*2 was 7.0% (8/115) and 35.7% (41/115), respectively (Figure 3A). Thus, *dam*2 was the most common among the *dam* homologs; in one third of the cases (12/41), it was part of an RM system.

The prevalence of *dcm*8 and *dam*3 was 26.1% (30/115) each. It was not possible to distinguish with PCR whether they occurred as a fusion protein or individually. The prevalence of *dcm* genes was as follows: 41.7% *dcm*1 (48/115), 22.6% *dcm*2 (26/115), 7.8% *dcm*3 (9/115), 3.5% *dcm*4 (4/115), 50.4% *dcm*5 (58/115), and 27.0% *dcm*6/7 (31/115). Forty-nine different combinations of *dcm* and *dam* genes were found (Supplementary Table S4). Solitary MTases were found in 97.4% (112/115) of the strains. An amount of 69.6% (80/115) of the strains carried more than one orphan MTase with a dominance of two and a maximum of four MTases per strain (Figure 3B). RM systems were present in 53.0% (61/115) of the strains; 9.6% (11/115) of the strains carried more than one RM system, and only one strain (8958) carried three RM systems (*dam*2, *dcm*2, and *dcm*6/7).

### 3.3. Transcription of M. hominis DNA MTases

**Figure 4:**
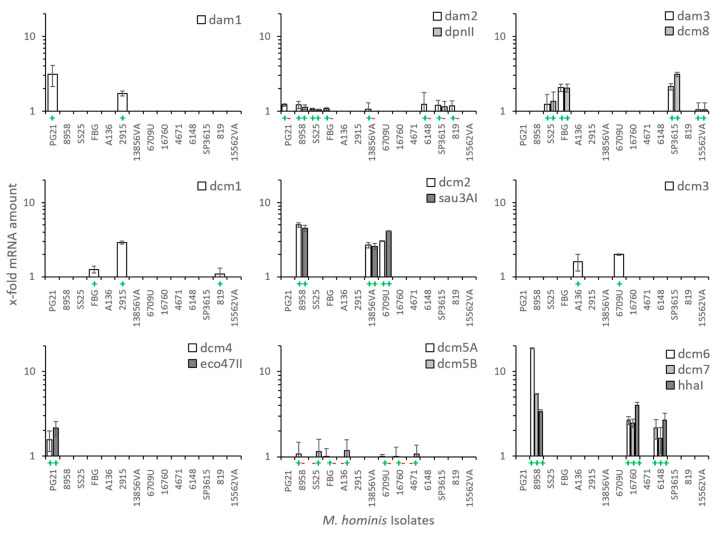
DNA MTase and REase transcripts in selected *M. hominis* strains. Relative transcript levels of each DNA MTase and, if associated, REase gene (rease) were calculated to the mean of three housekeeping genes (*gap*, mho_0150, and *lgt*). Presence of the respective genes is marked by (+); absence of next neighboured RM-genes is marked by (−). Bar graphs show the mean of the transcripts with the standard deviation of two independent cultures, each tested in duplicate.

In a selection of *M. hominis* strains, the expression of each DNA MTase was detected at the transcript level (Figure 4).

Strains without a respective MTase or REase gene were tested negative, and strains carrying the MTase or REase gene were tested positive. The RM system of *dcm*6/7 was highest expressed (4.8 (+/− 1.21)-fold increase) followed by RM-*dcm*2 (3.5 (+/− 0.61)-fold) and RM-*dcm*8/*dam*3 (1.7 (+/− 1.0)-fold). RM-*dam*2 (1.1 (+/− 0.68)-fold) and -*dcm*5 (A or B; 1.07 (+/− 0.9)-fold) were lowest transcribed. (Kruskal–Wallis *p*-value = 0.0063; for RM systems with more than one component, means of the gene expression of all components were calculated).

### 3.4. Conserved Domains of the Type II MTases

**Figure 5:**
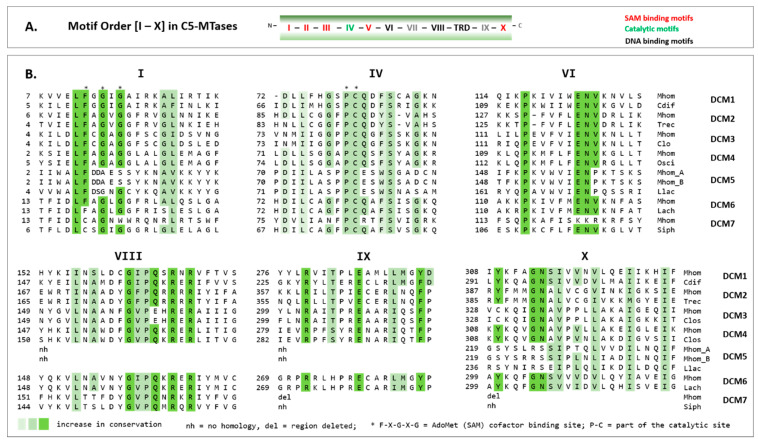
Alignment of conserved motifs of C5-cytosine MTases of *M. hominis*. (**A**) Schematic representation of motif order (I to X) in C5-cytosine MTases with SAM-binding motifs in red and catalytic motifs in green. (**B**) Consensus sequences of motifs I, IV, VI, VIII, IX, and X in DCM1-7 of *M. hominis* strains (Mhom) in multiple alignment with each highest homolog non-mollicutes species. Acronyms and homologies of the bacterial species are listed in Table 3.

As shown in Figure 5A five motifs (I–III, V, and X, coloured in red) are known to take part in the SAM-binding domain and one motif (IV, coloured in green) to be part of the catalytic domain in C5 MTases. Six motifs (I, IV, VI, VIII, IX, and X), which were more highly conserved, were analysed in more detail in DCM1 to DCM7 in a multiple sequence alignment (Figure 5B), each with a respective non-mollicutes homolog (Table 3).

In DCM1-4 and DCM6, the motif-dependent conservation was high, suggesting the functionality of these postulated MTases. In DCM5A and DCM5B, the SAM binding site F-X-G-X-G on motif I was not expressed, and the TRD-flanking motifs VIII and IX were too variant for detection; in DCM7, the TRD-downstream motifs IX and X were completely deleted, suggesting loss of enzymatic function of DCM5 (A and B) and DCM7.

**Figure 6:**
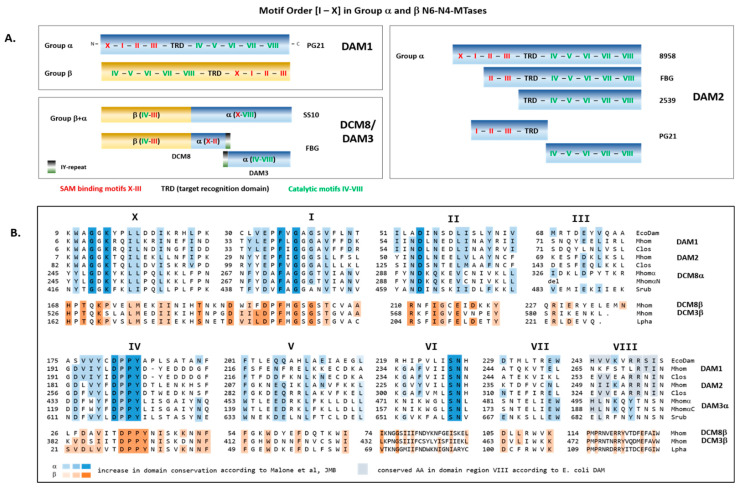
Alignment of conserved motifs of N-6-adenine and N-4-cytosine MTases of *M. hominis.* (**A**) Schematic representation of motif order (I to IX) in N-6-adenine and N-4-cytosine MTases. (**B**) Consensus sequences of motifs I to X in N-6-adenine MTases (DAM1, DAM2, and DCM8α/DAM3α) and N-4-cytosine MTases (DCM3β and DCM8β) of *M*. *hominis* strains (Mhom) in multiple alignment with each highest homologous non-mollicutes. Colour coding of conserved amino acids corresponds to that of Malone et al. [11]. Acronyms and homologies of the bacterial species are listed in Table 3.

N-6-adenine and N-4-cytosine MTases are subdivided into six groups based on group-specific motif conservations. DAM1 and DAM2 were assigned to belong to the α group with the SAM-binding motifs X, I–III (coloured in red) N-terminal to the TRD region and the catalytic motifs IV–VIII (coloured in green) C-terminal (Figure 6).

Deletions within the *dam*2 gene, which were always accompanied by the loss of the REase gene, affected loss of motifs X and I in strain FBG and of the whole SAM-binding domain in strain 2539. In *dam*1-positive strains, the *dam*2 pseudogene fragments encode a truncated motif I, motifs II and III with the N-terminal part of the TRD region (N-terminal fragment), and the catalytic motifs IV to VIII (C-terminal fragment). A DAM2 activity of both peptides as a protein complex was ruled out due to the lack of an overlap of both fragments and the deletion of 22 amino acids in the TRD region.

Based on the motif order and characteristics, DCM3 and DCM8/DAM3 were assumed to represent fusion proteins with a β-group N6-N4-MTase: the DCM3 protein, which is composed of an N-terminal C5-Mtase (AA 1–360) fused with a C-terminal β-group N6-N4-Mtase (AA 361–592). The DCM8/DAM3 protein of strain SS10 is composed of an N-terminal β group N6-N4-cytosine MTase (AA 1–237) with a C-terminal α group N-6-adenine MTase (AA 238–534). As shown in the scheme of Figure 6A for strain FBG as representative, the DCM8/DAM3 fusion protein was disrupted in a TA repeat region of the TRD region of the α group MTase in most strains (FBG, SS25, VO31120, SP3615), which leads to IY repeats at the breaking points. In these strains expression of an active β group MTase (DCM8), and an inactive α group N6-adenine MTase remnant (DAM3) was postulated. The Sanger sequencing of a PCR product, which was primed by dcm8F and dam3R to amplify the postulated TA repeat region, revealed a mixture of TA-repeat numbers in the strains tested (SS10, SP3615, FBG), which documented the expression of both full-length [(TA)_3n_] and disrupted [(TA)_3n(−1 or −2)_] DCM8/DAM3 protein variants in the same strain without indicating the prevalent variant.

As coloured in Figure 6B, the F-X-G-X-G stretch in motif I, which is important for SAM binding, and the D-P-P-Y stretch in motif IV, part of the catalytic site, were both highly conserved in DCM3β and DCM8β. Whether DCM3 and full-length DCM8/DAM3 express both MTase activities needs further investigation.

### 3.5. Putative Functions of the Type II MTases

In order to assign possible methylation specificity to the orphan MTases and RM systems, conserved domains and the function of the most homolog representatives in non-*M. hominis* mollicutes and other bacteria were considered (Table 4).

Due to the sequence homology of the adjacent REases to known restriction endonucleases, it was possible to predict the sequence motif of methylation for some RM-MTases: (G**^m^A**TC) for DAM2 with the associated *Dpn*II-homolog, cutting unmethylated (X│G**°A**TC); (GAT**^m^C**); for DCM2 with the associated *Sau*3AI-homolog, cutting unmethylated (X│GAT**°C**); (GGN**^m^C^m^C**) for DCM4 with the associated *Eco*47II-homolog, cutting unmethylated (G│GN**°C°C**) without certainty, and one of the C5-residues will be methylated; and (G**^m^C**GC) for DCM6/7 with the associated *Hha*I-homolog, cutting unmethylated (GCG│**°C**).

### 3.6. Bioinformatics on Methylation Profile of Nanopore Sequenced M. hominis Genomes

The Tombo script was adapted and run on the Nanopore sequenced *M. hominis* strains to analyse 6mA and 5mC in the above mentioned sequence motifs (5′-3′: CCWGG, GATC, GGNCC, and GCGC), motif GGATC and motifs published for other mycoplasmas (CATG, CG, CTAT, GAAG, GAGG, GGAG, GANTC, GATGC, and GCNGC) [26,28] and the reverse-complementary motifs. Methylation scores for all sequence motif variants (with W = A or T and N = A, G, C or T) are listed in the Supplementary Excel File: Tables S5 (5mC) and S6 (6mA). Nineteen isolates showed at least one sequence motif with methylation frequencies above 0.5 (Table 5). Forty-six of the seventy-four (5mC and 6mA) methylation motifs were not calculated positive; the others are listed with the pattern of *dam* and *dcm* genes in Table 5.

### 3.7. CCWGG Methylation

5′-CCWGG-3′ is the well-known target sequence of DCM in *E. coli* and other bacteria. Of the 22 Nanopore sequenced *M. hominis* strains, 9 were *dcm*1 positive even though methylation of 5′-CCWGG-3′ was only observed for strains 10936, 14352VA, and 18847. Additionally, the DNA of strain SP2565 showed methylated 5′-CCWGG-3′ motifs despite the lack of a *dcm*1 gene. An analysis of the methylation profile with respect to the sequence motif variants (W = A or T) revealed the highest methylation frequencies of 5′-C^m^CTGG-3′ followed by 5′-^m^CCTGG-3′ (Supplementary Table S5). The 6mA methylation of 5′-CC^m^AGG-3′ was observed in strains SP2565, SS10, VO31120, 18847, and 475 in descending order, indicating the presence of another, previously undiscovered 6mA DNA MTase. All CCWGG variants with respective 6mA and 5mC methylation scores are listed in Supplementary Tables S5 and S6 and shown for a selection of *dcm*1-positive and -negative isolates in Figure S3.

### 3.8. GCNGC Methylation

Exclusively in strains 10936, 14352, and 18847, a further sequence motif, 5′-GCNGC-3′, was found to be 5mC methylated. Tombo analysis of all sequence variants (N = A, G, C, or T) revealed the highest methylation scores in 5′-GCTG^m^C-3′ (0.9/0.8/0.8, respectively). Whether DCM1 performs an off-target 5mC methylation at this motif or a yet undetected 5mC-MTase is responsible for the methylation requires further investigation.

### 3.9. GATC Methylation

GATC is the target sequence motif of the well-studied 6mA MTase DAM (G^m^ATC) and a 5mC DNA MTase (GAT^m^C). Nanopore sequenced *M. hominis* strains carrying *dam*1 (12256U and SP10291) or full-length *dam*2 (14355VA, 7388VA, 942J, 475, SS25, and 8958) possessed G^m^ATC-methylated DNA. The methylation scores of strains carrying only *dam*2 remnants (marked with 0.5 in Table 5) were lower than 0.5. Strains 19791 and SP2565, lacking both *dam*1 and *dam*2 genes but carrying *dcm*2, showed methylation frequencies of 0.6 for G^m^ATC. In *dcm*2-deficient strains carrying *dam*1 or *dam*2, 6mA methylation scores of an enlarged motif (G^m^ATCC/GG^m^ATC) were calculated as higher than those of G^m^ATC (Figure S4). In total, 4 of the 22 Nanopore sequenced strains were *dcm*2-positive (12256U, 19791, 8958, and SP2565), which correlated with GAT^m^C methylation scores of 0.75, 0.88, 0.9, and 0.95, respectively.

### 3.10. CCTC Methylation

5mC methylation of the non-palindromic 5′-CCTC-3′/5′-GAGG-3′-double strand was observed in four of the 22 Nanopore sequenced strains (14352, 19791, A136, and VO31120) and correlated with a *dcm*3 presence. The methylation frequency was higher for the first than the second cytosine and near the background for the third. A 6mA methylation of the reverse complementary 5′-G^m^AGG-3′ motif was not detected.

### 3.11. GCGC Methylation

In 7 of the 22 Nanopore sequenced strains, 5mC methylations in the 5′-GCGC-3′-motif were detected, mainly at the first, but also at the second cytosine (MethScores about 0.9 and 0.5–0.6. respectively). As these strains all carried the *dcm*6/*dcm*7 RM system with a *Hha*I homolog REase (cutting GCGC), *dcm*6/*dcm*7 was assigned to the GCGC methylation.

### 3.12. Some Loose Ends: Methylated Sequence Motifs and MTases without Counterpart

As listed in Table 5, all but one of the other sequence motifs (GGNCC, CTTC, GGAG, GASTC) had methylation scores just above cut-off (0.5–0.6), which could indicate a high background rather than specific methylation. The methylation score for 6mA in motif CT^m^AT was higher than 0.6 in five strains with a maximum of 0.9 in strain 8958, suggesting the presence of an additional, yet undetected 6mA MTase.

No 6mA or 5mC methylation motif tested corresponded to the gene presence of *dcm*8/*dam*3 or *dcm*5 (A and B) in the analysed strains. Noteworthy, the three strains 10936, 14352VA, and 18847, which carried the 5mC methylated motifs CCWGG and GCNGC (see above), all carried *dcm*5A (18847) or *dcm*5B (10936, 14352VA) besides *dcm*1, but they had no other MTase gene in common.

### 3.13. Proof of Methylation Activities

**Figure 7:**
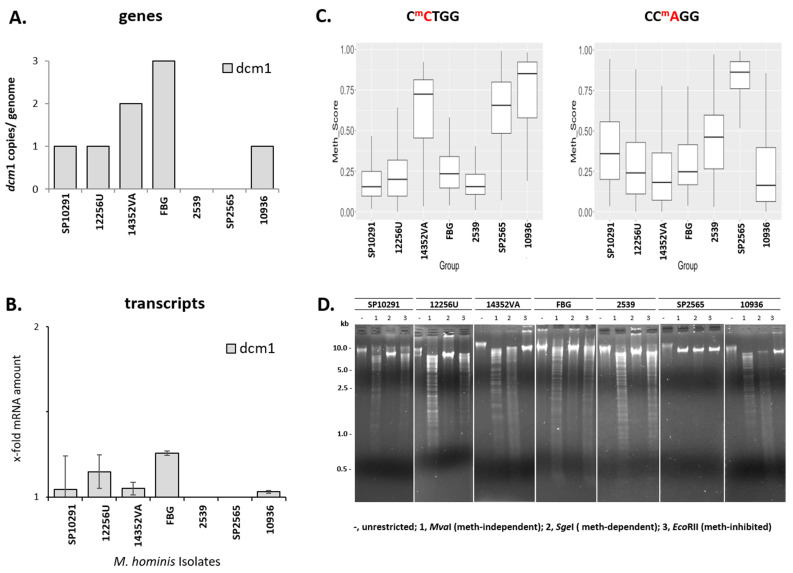
Proof of DCM1 activity in methylation sensitive restriction (MSR) analysis. (**A**) Number of *dcm*1 genes per genome. (**B**) Relative transcript levels of *dcm*1, calculated to the mean of three housekeeping genes (*gap*, mho_0150 and *lgt*) with the standard deviation of two independent cultures, each tested in duplicates. (**C**) Plots of methylation scores calculated with tailored Tombo script (CC^m^AGG Kruskal–Wallis chi-squared = 649.26, df = 6, *p*-value < 2.2 × 10^−16^, C^m^CTGG Kruskal–Wallis chi-squared = 843.05, df = 6, *p*-value < 2.2 × 10^−16^). (**D**) MSR analysis. Restricted DNA was separated on 1% (*w*/*v*) agarose gels.

Next, the 5mC methylation of CCWGG, which had been predicted by Tombo for some *dcm*1-negative isolates, and 6mA methylation of GATC for some *dam*1/*dam*2-negative isolates, were verified using a methylation sensitive restriction (MSR) approach. To this end, genomic DNA from seven *M. hominis* strains was first subjected to a *dcm*1 MSR analysis (Figure 7).

Five strains (SP10291, 12256U, 14532VA, FBG, and 10936) were *dcm*1 genes and transcript positive (Figure 7A,B), but only strains 14352VA and 10936 expressed DCM1 activity. The DNA of these strains, with calculated methylation scores ≥ 0.7, was cut by methylation-dependent restriction endonuclease *Sge*I but not methylation-sensitive *Eco*RII, demonstrating C^m^CTGG methylation (Figure 7D). The DNA of the *dcm*1-positive strain, SP10291, 12256U, and FBG, with calculated C^m^CTGG methylation frequencies <0.25, showed restriction patterns of non-methylated CCWGG, such as the *dcm*1-deficient strain 2539 (Figure 7D). Strain SP2565, which was also *dcm*1 gene- and transcript-negative (Figure 7A,B), had methylation scores of 0.7 for C^m^CTGG and 0.9 for CC^m^AGG (Figure 7C). As the methylation-sensitive restriction pattern of strain SP2565 correspond to its genetic *dcm*1 deficiency, a Tombo-calculated 5mC methylation score of 0.7 for C^m^CTGG was suggested to be based on a cross-detection of the 6mA methylation of CC^m^AGG (Methylation Score 0.9).

**Figure 8:**
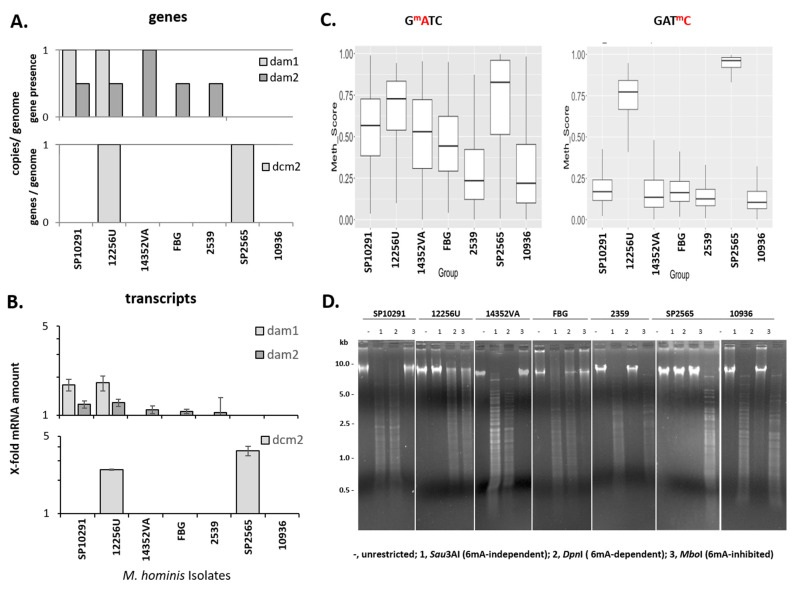
Proof of DAM1 and DAM2 activity in MSR analysis. (**A**) Presence of full-length genes was assigned a value of 1, truncated genes a value of 0.5, and absent genes a value of 0. (**B**) Relative transcript levels of *dam*1, *dam*2, and *dcm*2, calculated to the mean of three housekeeping genes (*gap*, mho_0150, and *lgt*) with the standard deviation of two independent cultures, each tested in duplicates. (**C**) Plots of methylation scores calculated with tailored Tombo (G^m^ATC Kruskal–Wallis chi-squared = 4365.2, df = 6, *p*-value < 2.2 × 10^−16^, GAT^m^C Kruskal–Wallis chi-squared = 10,077, df = 6, *p*-value < 2.2 × 10^−16^). (**D**) MSR analysis. Restricted DNA was separated on 1% (*w*/*v*) agarose gels.

Second, we took a closer look at the putative, Tombo-calculated 6mA methylation of G^m^ATC in strains with *dam*1- and *dam*2-negative background, testing the same strains as for *dcm*1 activity. As shown in Figure 8, presence of the *dam*1-, *dam*2-, and *dcm*2-genes (Figure 8A) was accompanied by the respective transcript evidence, even in the case of gene truncations (Figure 8B).

DNA of strains carrying *dam*1 (SP10291 and 12256U) or full length *dam*2 (14352VA) with calculated methylation frequencies >0.5 (Figure 8C) was cut by *Dpn*I demonstrating G^m^ATC methylation (Figure 8D). Methylation sensitive G^m^ATC restriction and Tombo-calculated methylation scores decreased from strain FBG (0.5) to 2539 (0.25), correlating with the degree of *dam*2 truncation (see Figure 6A).

The DNA of two analysed strains (12256U and SP2565) was not restricted by *Sau*3AI, the endonuclease originally intended as a control for the 6mA methylation-independent cutting of GATC. This finding could be explained by the fact that *Sau*3AI restriction is sensitive to 5mC methylation of a GAT^m^C presence and that the respective strains all harboured *dcm*2 and a *Sau*3AI homolog REase gene as an RM system (see Table 4). The assumption that DCM2 methylates GAT^m^C was further supported by restriction with 5mC methylation-independent *Dpn*II (GAT°C) but not 5mC methylation-inhibited *Sau*3AI (GAT^+^C) (Figure S5).

The high 5mC methylation scores of GATC in strains SP2565 and 12256U were also reflected in 6mA methylation of the same motif. SP2565 does not carry *dam*2 (Figure 8A) and appears in methylation-sensitive restriction unmethylated in 6mA of the GATC motif. The calculation of a false-positive 6mA methylation value by Tombo due to the strong methylation of the neighboured 5mC in GATC can also be assumed here (as formerly for *dcm*1).

**Figure 9:**
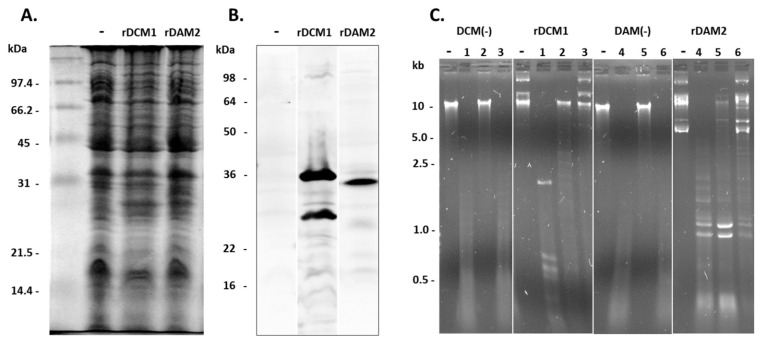
rDCM1 and rDAM2 expression led to respective methylation activity of transformed *E. coli*. Coommassie blue staining of total protein (**A**) and immunostaining of histidine-tagged proteins (**B**) in cell lysate of *dam*-/*dcm*-deficient *E. coli* (−), transformed by pcDNA3.1-(codon-optimized) *dcm*1 of PG21 (rDCM1) or –(codon-optimized) *dam*2 of 8958 (rDAM2). (**C**) MSR analysis of the respective genomic *E. coli* DNAs; unrestricted (DCM(−) and DAM(−)) or restricted with *Mva*I (1), *Sge*I (2), and *Eco*RII (3) for rDCM1 clone and restricted with *Sau*3AI (4), *Dpn*I (5), and *Mbo*I (6) for rDAM2 clone.

As most of the analysed strains carried more than one family member of *dam* or *dcm* homologs, the final evidence of C^m^CWGG and G^m^ATC methylation activity was elucidated against a *dam*-/*dcm*-negative background. The *dam*-/*dcm*-deficient *E. coli* was transformed with *dcm*1 or *dam*2 expression plasmids and heterologous expression proven by tetraHis-staining in Western blotting (Figure 9A).

The recombinant proteins, rDCM1 and rDAM2, labelled with histidine at the C-terminal end were expressed at full length, but rDCM1 also showed an equally strong degradation product (Figure 9B). Methylation of the genomic DNA of rDCM1- and rDAM2-expressing *E. coli* clones (in contrast to plasmid-free DCM(−) and DAM(−)) was documented through methylation-sensitive restriction analysis as done before (Figure 9C). Cleavage of the *E. coli* DNA by the methylation-dependent restriction enzyme *Sge*I (rDCM1 (lane 2)) and *Dpn*I (rDAM2 (lane 5)) demonstrated the presence of methylated C^m^CWGG- and G^m^ATC-motifs and gave the final proof of DCM1 and DAM2 methylation activity, respectively.

### 3.14. Correlation between the Presence of DNA MTases and Mobile Genetic Elements

**Figure 10:**
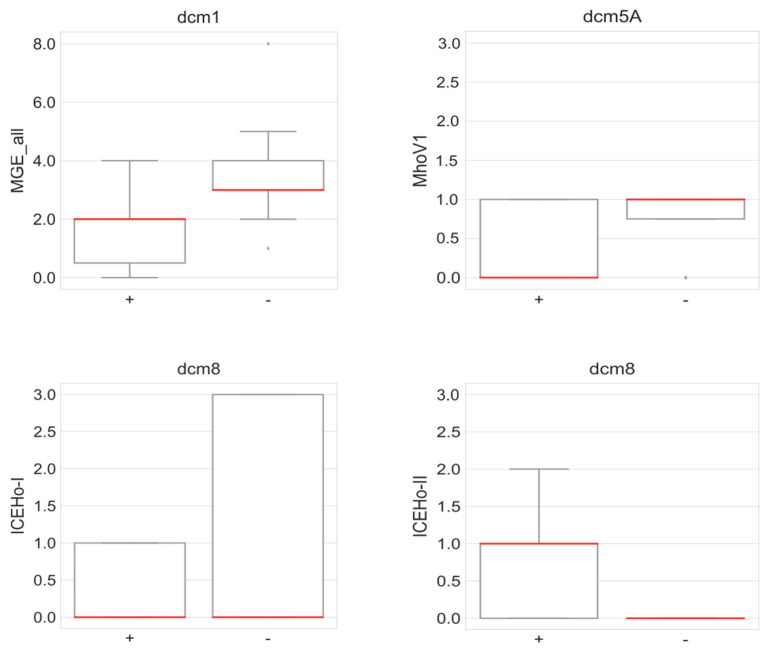
Boxplots of MTases and MGEs. Presence of selected MTases (+ present, − not present) correlated with number of selected MGEs, according to the significance in Pearson’s R correlation analysis (see Figure S6). Median bar is shown in red. (Mann–Whitney U MGE_all vs. dcm1 *p* = 0.0001; MhoV1 vs. dcm5A *p* = 0.4677; ICEHo-I vs. dcm8 *p* = 0.2818; ICEHo-II vs. dcm8 *p* = 0.0930).

Next, we had a look on a putative correlation between presence of DNA MTases (solitary or RM systems) and mobile genetic elements (Supplementary Figure S6). Since active RM systems should prevent the uptake of foreign DNA, a negative correlation between RM and MGE was expected; however, neither RMs and MGEs in total, nor each combination of an individual RM and MGE correlated statistically significantly. With regard to the solitary MTases, correlations were found (Figure 10).

The co-occurrence of *dcm*1 and MGEs (*p* = 0.00001) was caused by a perfect correlation between *dcm*1 and ICEHo-I (*p* < 0.0001), which was not surprising since *dcm*1 is a conserved gene of the ICEHo-I element [31]. In addition, the presence of *dcm*1 and ISMhom1 was also positively correlated (*p* < 0.005), and three further combinations were of statistical significance (Figure 10). We calculated a positive correlation between *dcm*5A and MhoV1 (*p* < 0.05) and *dcm*8/*dam*3 and ICEHo-I (*p* < 0.1) and a negative correlation between *dcm*8/*dam*3 and ICEHo-II (*p* < 0.05). No further correlations calculated significant with restriction on the methylation active *dcm*1 or *dam*2 strains (Figure S6).

## 4. Discussion

This is to the best of our knowledge the first approach to use a tailored Tombo script for motif driven analysis of 5mC and 6mA methylation frequencies in Nanopore sequenced *M. hominis* genomes. Our analysis benefited from the availability of completely resolved isolate genomes, enabled by the ability of the Oxford Nanopore technology (ONT) to sequence long fragments of DNA and thus generate sufficiently long reads to span repetitive genomic regions and from the ability to directly call methylated bases from raw Nanopore sequencing data. In the context of our study, the long-read sequencing aspect as well as methylation calling capability of the technology have to be counted as the main advantages of the Nanopore sequencing technology. In addition, native ONT sequencing requires no complex sample processing steps; no PCR amplification, which could result in a biased dataset; and involves no degradation and loss of input material. However, one limitation of the present study is that the Nanopore-based methylation analysis presented here could be extended and potentially improved in multiple ways. First, our analysis was based on site-specific methylation scores computed with the generic 5mC and 6mA models implemented in Tombo; in addition, the “de novo” workflow available in Tombo could be applied, which enables us to combine an analysis of raw signal data with a motif discovery tool, such as the MEME Suite, to detect sequence contexts putatively affected by methylation. Second, the analysis presented here was based on a list of pre-defined sequence motifs from the literature, and it did not take into account potential interactions between motifs (potentially relevant, e.g., when an individual C base in the analyzed genome is part of two different motifs); a follow-up analysis could implement a more comprehensive statistical model of site-specific methylation scores with support for sequence motif interaction effects and novel motif discovery (e.g., in a generalized linear model framework). Third, alternative methods for determining site-specific methylation scores from Nanopore data could be applied, such as the Hidden Markov Model-based method nanopolish [39] or the more recently published Nanodisco [40], which is also able to detect 4mC, 5mC, and 6mA in bacteria and microbiome data. Fourth, a comprehensive training dataset comprising Nanopore sequencing data from mycoplasma isolates with known or biotechnologically modified methylase activities could enable re-training or refinement of the methylation detection methods’ underlying models, enabling methylation calling in mycoplasma genomes with improved accuracy.

In the study by Dordet-Frisoni, a combination of SMRT-seq and Illumina bisulphite sequencing was used to detect all methylation sites [26]. They were able to draw a comprehensive picture of the methylome for *M. agalactiae*. However, some enzyme assignments were missing, as in our study, and for a final evidence of the methylation capacity of an MTase, testing against an otherwise MTase-free background seems to be essential due to the large number of different MTases per isolate. In this study, we present the methylation-sensitive restriction approach as principle proof of methylation activity of DCM1 (C^m^CWGG) and DAM1 and DAM2 (G^m^ATC) as well.

Based on the bioinformatics approach of this study, a large family of *dam* and *dcm* homolog type II DNA MTase genes were identified in *M. hominis.* Due to the phylogenetic clustering of DNA MTases of *M. hyorhinis* [28] and *M. agalactiae* [26] with known methylation specificities with *M. hominis* MTase homologs (Supplementary Figure S2). The methylation motifs could be confirmed for the MAGa2700 homologs DAM1 and DAM2 (G**^m^A**TC) and the Mhy3 homolog (AEX14156.1) DCM2 (GAT**^m^C**), specified for the MAGa3950 homolog DCM4 (GGN**^m^C**C) and hypothesized for MAG6680 homolog DCM8 (G**^m^A**NTC) and MAGa4250 homolog DCM6/7 (G**^4m^C**NGC). **^m^C**pG-methylation activity, which was detected in *M. agalactiae* (MAGa4470, MAG4480) and *M. hyorhinis* (Mhy1; AEX13846), was not evident in the *M. hominis* isolates tested.

In *M. hominis*, five solitary MTases (DAM1, DCM8/DAM3, DCM1, DCM3, DCM5) and four RM systems (*Dpn*II-DAM2; *Sau*3AI-DCM2, *Eco*47II-DCM4, and *Hha*I-DCM6/7) could be identified, whose strain-specific presence generated a variety of MTase combinations, suggesting an impact on the epigenetic heterogeneity of this species. Not only the presence or absence of a DNA MTase, but also strain-dependent expression affected the methylation profile. Whenever *dam*1, *dcm*2, *dcm*3, or *dcm*6/7 were found, the respective methylation of G^m^ATC, GAT^m^C, C^m^CTC, and G^m^CGC occurred while the expression of DCM1 activity, shown to preferentially methylate C^m^CTGG, did not occur in all *dcm*1 bearing strains. As *dcm*1 transcripts were found in each *dcm*1 positive strain, the regulation of DCM1 activity must occur on a (post-) translational level. Remarkably, *dcm*1 was the only MTase gene of *M. hominis* being transported by an integrative and conjugative element, ICEHo-I, whose expression might require other stimuli than liquid culturing, such as a polymicrobial presence, mating, or others. However, no methylation mapping on the genome sequence was done so far. 5mC and 6mA mapping would perhaps help to assess self-regulatory methylations and to understand the on/off of DCM1 activity in *M. hominis*.

For two MTases, DCM5 and DCM8/DAM3, no methylation motifs could be assigned. Neither the presence of DCM5A nor of DCM5B (or of both) correlated with one of the analysed methylation motifs. This is in accordance with the findings in *M. agalactiae*, where the DCM5-homolog MTase MAG4030 was predicted to methylate 5mC, even though no methylation motif could be identified [26]. Although an undetected methylation motif might account for this, it is of course also conceivable that it reflects the inactivity of these MTases, which originated in lack of essential MTase domain structures, as written above. However, the statistically significant calculated occurrence of DCM5A with an MGE (MhoV1) could also indicate functionality, although the small sample size in the correlation analysis must be considered a limitation.

A DCM8 homolog was detected in *M. agalactiae* (see Figure S2). This MTase, MAG6680, was characterized as a solitary type II MTase targeting 6mA of the GANTC-motif. However, the presence of *dcm*8/*dam*3 did not correlate with a low-threshold methylation of G^m^ASTC (S = G or C) detected in some of the analysed *M. hominis* strains, so its methylation motif is probably a different one. The results of a Phyre2 analysis of the β group MTase part of DCM3 and DCM8 revealed a structural homology to the M1.hpyavi-sam complex (with 58% and 54% amino acid identity and 100% confidence to template c5hfjF:PDB). The main substrate specificity of this DNA N6-adenine methyltransferase was G**^m^A**GG in most *H. pylori* strains [41]. Interestingly, the DCM3 of *M. hominis* was proposed to methylate the reverse complementary sequence motif, **^m^C**CTC and C**^m^C**TC, and its homolog in *M. agalactiae*, J7894_00205, was shown to methylate **^m^C**CTC and G**^m^A**GG. That DR-C0020 (acc.-no WP_034351354.1), a protein of *Deinococcus radiodurans*, was characterized as SAM-dependent C-5 methylase, although sharing amino acid sequence homologies to 4mC methylases [42], suggested that the prediction of methylation specificity (5mC or 6mA), which is only based on a global sequence similarity, should be handled with care and needs confirmation. Moreover, it has already been described that an MTase can change its target specificity [43]. With this knowledge, cross reactivity of 6mA to 5mC, as observed for CCAGG and GATC in Tombo analysis, always require a more detailed analysis to distinguish between a cross-reaction or multifunctionality in methylation.

Most of the type II MTases found in *M. hominis* have homologs in other bacteria, but their role in virulence and defence has not yet been studied comprehensively. In an analogy to MTases in other bacteria, the RM systems of *M. hominis* may mainly serve as primitive defence systems, and the solitary MTases regulate cellular processes. The correlation analysis of this study revealed that the RM systems of *M. hominis* are not obviously drivers of horizontal gene transfer as hypothesized in the study of Dordet-Frisoni et al. for *M. agalactiae* [26]. Two MTases, DCM3 and DCM5, may also take part in defence mechanisms, as the neighboured genes putatively encode (HNH-) endonucleases, family members of which are known to catalyse DNA hydrolysis.

In *M. agalactiae*, *dam* occurred as part of an RM system, but in *M. hominis*, *dam* occurred solitary (*dam*1) or as part of an RM system (*dam*2). As the solitary DAM in *E. coli* influences gene regulation by GATC methylation and by competing with regulatory proteins in their binding to promotor regions [44], DAM1 of *M. hominis* may influence gene regulation while DAM2 may take part in defence as a primary RM function. However, why do both DNA MTase systems exist in the genome-minimized *M. hominis*, each methylating the same G^m^ATC motif? Murphy et al. hypothesized [45] that an orphan MTase, methylating the same sequence motif as the MTase of an RM system, enables the survival of the organism in the case of a segregational loss of the (plasmid- or MGE-encoded) RM system. This theory fits well with our findings that *dam*1 was only present in strains with total *Dpn*II gene loss and a loss of DAM2 function. However, as the DAM2-RM system is affected by strain-dependent deletions of the REase gene, it would be of interest to analyze whether DAM2 function will change to regulatory functions, and it remains to be clarified whether both DAM MTases, which significantly differ in size and sequence, methylate the same GATC sites in the genome.

Furthermore, the role of DCM(1) remains ill-defined. A partial methylation of CCWGG sites was observed in *E. coli* cultures of a mid-exponential phase [46], and DCM heterologous was expressed in *E. coli* improved bacterial fitness during stationary phase at low temperatures in contrast to the DCM-free *E. coli.* Besides the involvement in the upregulation of ribosomal activity during the stationary phase [47], DCM was characterised to protect against the uptake and manifestation of a parasitic RM system [48], but also to be associated in MGE rearrangement and TN3 transposition [49]. DCM1 of *M. hominis* as conserved part of the MGE ICEHo-I is probably most involved in the last one. A general negative correlation between number of defence islands (RM systems) and ICE presence, attested in *M. agalactiae*, was not calculated significant in *M. hominis*. That *M. hominis* strain 8958 harbours three RM-systems, but is reduced in the number of MGEs, was not a generalised phenomenon observed in the long-read sequenced *M. hominis* strains. Only certain solitary MTase genes correlated with the presence or absence of certain MGEs: *dcm*5A correlated with MhoV1 presence, *dcm*8/*dam*3 with ICEHo-II absence, and weakly with ICEHo-I presence; suggesting that the methylation activity of DCM8/DAM3 (although of unknown motif) affects defence of the respective ICEHo uptake.

The DCM8/DAM3 fusion protein was shown to be affected by disruption within the DAM3 TRD region due to intra-strain variant numbers of a (TA) repeat. This had initially gone undetected in Nanopore and Illumina sequencing but has later been resolved by Sanger sequencing (of cloned PCR-products) in the *dcm*8/*dam*3-positive strains tested. In strain FBG, *dcm*8/*dam*3-variants with 6 to 12 TA-repeats were found, leading to full-length DCM8/DAM3 with an even TA repeat number but to an incomplete/fragmented protein because of a frameshift to stop codons with an odd TA repeat number. Short sequence repeats (SSRs) are indicative for phase-variable expression, and their gain or loss was suggested to develop during DNA replication by slipped-strand mispairing [50]. Depending on their length and multiplicity, they can lead to frameshift and consequently a premature translation stop or alter the secondary structure and specificity. So far, SSRs were characterized in a magnitude of surface proteins, affecting surface diversity and thereby improving a host immune escape, but they were also found in type I and type III RM systems of various pathogens [51]. In contrast to type II RM systems, characterized in this study, type I and type III RM(S) systems have their own domain (S) for substrate specificity. If SSRs were located at the 5′-end of M or S genes, repeat gain or loss led to an ON/OFF expression of the genes [51,52,53]. In *Streptococcus suis*, the OFF-mode of the type III DNA MTase ModS2-domain was shown to impact growth and ampicillin resistance. If SSRs were located within the gene (e.g., between both TRD domains of type I *hsd*S) repeat gain or loss led to expression of full-length or truncated protein [53,54] or (due to loss or gain of amino acid repeats) a protein with new specificity. With the detection of a TA-repeat region in the DAM3-TRD region of DCM8/DAM3, the copy numbers of which affect full-length expression of both MTase parts or only the expression of DCM8 with DAM3 remnants, this fusion protein is postulated as the first type II DNA MTase expressing phase-varions. Future studies should be done to demonstrate expression of the MTase-varions and to characterize their impact on defence and virulence of *M. hominis*.

## 5. Conclusions

The type II DNA methyltransferases of *M. hominis* characterized in this study correspond to homologs described in other bacteria. Unique for *M. hominis* are two MTases (the solitary DAM1 and the RM system based DAM2) methylating the same motif, G^m^ATC, and the postulated intra-strain specific phase-varions of an MTases-fusion (DCM8/DAM3). The repertoire of solitary and RM-based MTases was strain specific and demonstrated gain and loss of genes in *M. hominis*. From an evolutionary point of view, the gain of virulence- or defence-associated genes, such as the DNA MTases, seems to counteract the parasite-induced minimization of mycoplasma genomes to contribute to the heterogeneity of this human facultative-pathogen, *M. hominis*.

## Figures and Tables

**Table 1:** Primers used for qPCR screening and mRNA quantification.

Target	Forward Primer	Sequence (5′-3′)	Reverse Primer	Sequence (5′-3′)	Amplicon (bp)
gap	gap-F	GCAGGCTCAATATTTGACTCACT	gap-R	GATGATTCATTGTCGTATCATGC	95
hitA	hitA-F	TTGAGGCACAGCAATAGC	hitA-R	AAGGCTTAGGTAAGGAATGATTAG	83
lgt	lgt-F	TGAAATTGATTACGTCCAGGAA	lgt-R	CCGAAACGAATTATTCCATAATAAAC	68
mho_0150	mho150-F	CAAGGTTCAAATGATATTGCAAAG	mho150-R	CAATTCCATATCCAATAGGAACAA	94
dam1	dam1-F	ATGGGCAGGCGGAAAAC	dam1-R	GAATACTGCTCCTCCACCAATAAA	118
dam2	dam2_F	TTGTAAAATGAGCAGGTGGHARGACACA	dam2-R	CTAAAAAGTAATGAACCYCCRCCAATAAATG	115
R.dam2	R_dam2-F	AATGAGGTTGCCAGAAGTTATAGRA	R_dam2-R	CCRYAACCATCAGTAAATCAAASAAATG	89
dam3	dam3-F	TGAAAATGCAAGAAACAAGAGAA	dam3-R	GCACCCGAAATTAAGTATGGA	67
R.dam3	R_dam3-F	CATTGCCAATTTTTAAGGTGGATAAT	R_dam3-R	TGTTTTAGGGCAATGTATTTTTCTGAT	148
dcm1	dcm1-F	CACGGATCTCCTTGTCAAGAT	dcm1-R	TGTTTCCCACAATAAACTACTTCG	91
dcm2	dcm2-F	TTGCTTTGCGGTGGTTTTC	dcm2-R1	ACTCCTTTTTTRCCTTYAATACCTTTTTC	86
R.dcm2	R_dcm2-F	AAAAGGAGCGAATAGCAHAGTT	R_dcm2-R	GAGGAGAATAAGACGGCAAGTT	140
dcm3	dcm3-F	CGAAGAAGATGGGCGTAAATATG	dcm3-R	ACGCGGATCATCTAAACCTAAATT	92
dcm4	dcm4-F	GCGTGATATCGACCCAAATGT	dcm4-R	ATTCTACGAAGGATTCCAGTTCGT	87
R.dcm4	R_dcm4-F	CATTCGAATGATGAGTGTTTTTTAG	R_dcm4-R	GCCTTGCAGTGATTTCCATTTAC	95
dcm5A	dcm5A_F	ACCAGATATTATTTTAGCCTCACC	dcm5A_R	ATCGAACGCAGCATTTTG	78
dcm5B	dcm5B_F	GGTGGAAAAATGCTACGCTCT	dcm5B_R	ATCTGCCTCTTTCTTTTTGCTCA	133
dcm6	dcm6-F	TACGTACAGCCACAAATCAATCAG	dcm6-R	CAAAAACACCACCCCCATAAG	219
R.dcm6/7	R_dcm6/7-F	AAACGAAGCGGCTCATCCAG	R_dcm6/7-R	AAAACTTTATCGCCATCATCAGA	110
dcm7	dcm7-F	GTAATTGGTGGCGGCAGAATAG	dcm7-R	CATTAAGTCATAAGTTCGCTTGCTAA	98
dcm8	dcm8-F	ACCGGTAGAGTTAATGGAAAAA	dam8_R	TTAAGGCCGCAACRCAAGTC	104

**Table 2:** Restriction endonucleases with respective recognition sequence used for MSR analysis.

	MTase-	Restriction Enzyme ^a^		
Mtase	Motif	Cut1: °X	Cut2: ^m^X	Cut3: ^+^X
DCM1	C^m^CWGG	*Mva*I °C°CWGG	*Sge*I ^m^CNNGN_9_	*Eco*RII ^+^C^+^CWGG
DCM2	GAT^m^C	*Dpn*II G^+^AT°C	-	*Sau*3AI G°AT^+^C
DAM1	G^m^ATC	*Sau*3AI G°AT^+^C	*Dpn*I G^m^AT^+^C	*Mbo*I G^+^AT°C
DAM2				

^a^ Restriction: independent on methylation of amino acid (AA) X (°X), requires methylation of AA X (^m^X), or is inhibited by methylation of AA X (^+^X); respective AA X is marked in red.

**Table 3:** DAM and DCM proteins of *M. hominis* and highest homologous non-mollicutes species.

*M. hominis* DNA MTase				Highest NON-MYCOPLASMA Homologe of Non-Redundant Protein Sequences (nr) by BlastP						
Name	Reference Strain	Length (AA)	Accession	Acronym	Name	Query Cover	E Value	Identity (%)	Length (AA)	Accession
dcm1	FBG	333	QKX31507.1	Cdif	*Clostridioides difficile*	98%	2.00 × 10^−73^	44.12%	321	HBE9891886.1
dcm2	8958	414	QKX36700.1	Trec	*Treponema rectale*	98%	0.0	74.75%	412	QOS39629.1
dcm3	VO31120	592	QKX39759.1	Clos	*Clostridia bacterium*	99%	0.0	80.38%	596	MCD8040040.1
dcm4	PG21 (ATCC23114)	337	CAX37170.1	Osci	*Oscillospiraceae bacterium*	92%	6.00 × 10^180^	80.06%	352	MBP0955721.1
dcm5A	475	256	QKX38638.1	Llac	*Lactococcus lactis*	94%	2.00 × 10^−41^	39.93%	268	WP_118263419.1
dcm5B	A136	253	QKX37494.1	Llac	*Lactococcus lactis*	94%	2.00 × 10^−41^	40.30%	268	WP_118263419.1
dcm6	8958	325	QKX36719.1	Lach	*Lachnospira* sp.	99%	0.0	82.10%	330	MBQ4284079.1
dcm7	8958	213	QKX36723.1	Siph	*Siphoviridae* sp.	82%	1.00 × 10^−49^	50.56%	610	DAG15160.1
dam1	PG21 (ATCC23114)	442	CAX37613.1	Clos	*Clostridia bacterium*	97%	0.0	62.59%	439	MCR4661536.1
dam2	8958	282	QKX36937.1	Clos	*Clostridia bacterium*	98%	4.00 × 10^−93^	58.06%	355	MBO4694934.1
dcm8	SS10 (AA5-233)	534	QKX39057.1	Lpha	*Lactococcus phage*	42%	9.00 × 10^−79^	55.46%	227	YP_009904999.1
dam3	SS10 (221-529)	534	QKX39057.1	Sube	*Streptococcus uberis*	57%	1.00 × 10^−62^	41.05%	709	WP_154631601.1

**Table 4:** Highest Hits of Conserved Domains in solitary MTases and RM systems of *M. hominis*.

*M. hominis* Proteins			Highest Hit of Conserved Domains				
Name *	Isolate	Length (AA)	Name	Accession	Description	Interval (AA x-y)	E-Value
DAM1	PG21	442	dam	TIGR00571	DNA- adenine methylase (dam)	1–280	5.25 × 10^−58^
DCM1	FBG	333	Cyt_C5_DNA_methylase	cd00315	Cytosine-C5 specific DNA methylase	1–325	9.29 × 10^−27^
DAM2	8958	282	dam	TIGR00571	DNA- adenine methylase (dam)	2–274	6.16 × 10^−61^
R.DAM2	8958	291	*Dpn*II	pfam04556	DpnII restriction endonuclease	5–278	8.47 × 10^−98^
DCM2	8958	414	dcm	TIGR00675	DNA-methyltransferase (dcm)	7–407	1.32 × 10^−68^
R.DCM2	8958	463	*Sau*3AI_N-like	cd22356	N-terminal catalytic domain of Sau3AI	22–208	1.34 × 10^−83^
			*Sau*3AI_C-like	cd22355	C-term. allosteric effector domain of Sau3AI	243–459	4.31 × 10^−65^
DCM6	8958	325	DNA-methylase	pfam00145	C-5cytosine-specific DNA methylase	29–321	5.24 × 10−^107^
R.DCM7	8958	262	*Hha*I-like	cd21834	(Restriction) endonuclease	3–261	5.79 × 10^−102^
DCM7	8958	213	dcm	TIGR00675	DNA-methyltransferase (dcm)	47–186	4.08 × 10^−41^
DCM4	PG21	337	Cyt_C5_DNA_methylase	cd00315	Cytosine-C5 specific DNA methylase	24–330	1.81 × 10^−81^
R.DCM4	PG21	217	*Eco*47II	pfam09553	EcoR47II restriction endonuclease	7–211	3.76 × 10^−110^
DCM3	VO31120	592	dcm	COG0270	Site-specific DNA-cytosine methylase	1–355	2.73 × 10^−85^
			N6_N4_MTase	pfam01555	DNA methylase	383–584	4.37 × 10^−46^
up_DCM3	VO31120	409	HNH	pfam01844	HNH endonuclease	310–363	4.74 × 10^−8^
DCM5A	475	256	Cyt_C5_DNA_methylase	cd00315	Cytosine-C5 specific DNA methlyase	14–83	6.58 × 10^−3^
DCM5B	A136	253	Cyt_C5_DNA_methylase	cd00315	Cytosine-C5 specific DNA methlyase	14–85	2.14 × 10^−3^
down DCM5	475	979	DUF1524	pfam07510	HXXP-motif protein. similar to His-Me finger endonuclease	841–915	9.59 × 10^−03^
DCM8	SS10	534	N6_N4_Mtase	pfam01555	DNA methylase	27–227	3.38 × 10^−46^
DAM3			Dam	COG0338	site-specific DNA-adenine methylase	239–530	1.50 × 10^−21^

* R.DAM/DCM, restriction endonuclease of DAM/DCM; up_/down_DCM3, protein encoded by the gene upstream/downstream of the DCM3 gene.

**Table 5:** Methylation scores of variant nucleotides in the recognition sequences of selected motifs.

MTase Gene	Motif *	10936	18847	19791	21127	12256U	14352VA	7388VA	7447VA	942J	475	A136	SS10	SS25	VO31120	SP2565	SP10291	8958	FBG	2539
dcm1		1 **	2	1	1	1	2	0	0	1	0	0	0	0	0	0	1	0	3	0
	**C**CTGG	0.8	0.6	0.2	0.2	0.2	0.6	0.2	0.1	0.1	0.2	0.2	0.2	0.2	0.3	0.6	0.2	0.2	0.2	0.2
	C**C**TGG	0.9	0.6	0.2	0.2	0.2	0.7	0.2	0.1	0.1	0.2	0.2	0.2	0.2	0.3	0.7	0.2	0.1	0.2	0.2
	**C**CAGG	0.7	0.3	0.1	0.1	0.2	0.6	0.1	0.1	0.1	0.2	0.2	0.2	0.2	0.2	0.3	0.2	0.2	0.2	0.2
	C**C**AGG	0.6	0.3	0.1	0.2	0.2	0.6	0.2	0.1	0.1	0.2	0.2	0.2	0.2	0.3	0.4	0.2	0.2	0.2	0.2
dam?	CC**A**GG	0.2	0.5	0.3	0.3	0.2	0.2	0.3	0.4	0.3	0.5	0.4	0.6	0.4	0.6	0.9	0.4	0.5	0.2	0.5
																				
dcm?	G**C**AGC	0.9	0.8	0.2	0.3	0.3	0.8	0.3	0.2	0.1	0.1	0.2	0.3	0.3	0.4	0.3	0.2	0.3	0.4	0.2
	G**C**TGC	0.9	0.8	0.2	0.3	0.3	0.8	0.3	0.1	0.1	0.1	0.2	0.2	0.2	0.4	0.3	0.1	0.1	0.3	0.1
	GCTG**C**	0.7	0.5	0.2	0.3	0.3	0.6	0.3	0.3	0.2	0.3	0.4	0.4	0.4	0.4	0.3	0.3	0.3	0.3	0.3
	G**C**GGC	0.6	0.3	0.3	0.3	0.3	0.6	0.3	0.2	0.3	0.3	0.3	0.3	0.3	0.3	0.3	0.2	0.6	0.3	0.3
	G**C**CGC	0.8	0.4	0.2	0.3	0.2	0.4	0.2	0.3	0.2	0.2	0.2	0.2	0.2	0.2	0.2	0.2	0.3	0.2	0.2
	GC**C**GC	0.7	0.3	0.2	0.2	0.2	0.5	0.2	0.2	0.2	0.2	0.2	0.2	0.2	0.2	0.2	0.2	0.3	0.1	0.2
	GCCG**C**	0.2	0.2	0.2	0.2	0.2	0.2	0.3	0.2	0.3	0.4	0.3	0.3	0.3	0.4	0.3	0.3	0.4	0.3	0.3
																				
dam1		0	0	0	0	1	0	0	0	0	0	0	0	0	0	0	1	0	0	0
dam2		0	0.5 **	0	0	0.5	1	1	0.5	1	1	0	0	1	0	0	0.5	1	0.5	0.5
	G**A**TC	0.2	0.2	0.8	0.2	0.7	0.5	0.5	0.3	0.5	0.6	0.2	0.2	0.6	0.2	0.8	0.6	0.9	0.4	0.2
	G**A**TCC	0.3	0.3	0.6	0.2	0.6	0.7	0.7	0.5	0.7	0.7	0.2	0.3	0.7	0.3	0.6	0.6	0.8	0.5	0.3
	GG**A**TC	0.2	0.3	0.7	0.2	0.7	0.6	0.7	0.4	0.7	0.7	0.2	0.2	0.7	0.2	0.6	0.6	0.9	0.5	0.2
																				
dcm2		0	0	1	0	1	0	0	0	0	0	0	0	0	0	1	0	1	0	0
	GAT**C**	0.1	0.1	0.9	0.1	0.8	0.1	0.1	0.1	0.1	0.2	0.1	0.1	0.2	0.1	1.0	0.2	0.9	0.2	0.1
	GAT**C**C	0.1	0.1	0.9	0.1	0.8	0.2	0.1	0.1	0.1	0.2	0.2	0.2	0.2	0.2	1.0	0.2	1.0	0.2	0.1
	GATC**C**	0.1	0.1	0.9	0.1	0.8	0.2	0.1	0.1	0.1	0.2	0.2	0.2	0.2	0.2	1.0	0.2	1.0	0.2	0.1
	GGAT**C**	0.1	0.1	0.9	0.1	0.8	0.2	0.1	0.1	0.1	0.2	0.2	0.2	0.2	0.2	1.0	0.2	0.9	0.2	0.2
																				
dcm3		0	0	1	0	0	1	0	0	0	0	1	0	0	1	0	0	0	0	0
	**C**CTC	0.1	0.1	0.5	0.1	0.1	0.5	0.1	0.1	0.1	0.2	0.8	0.2	0.2	0.8	0.2	0.2	0.2	0.2	0.2
	C**C**TC	0.1	0.1	0.5	0.1	0.1	0.5	0.1	0.1	0.1	0.2	0.7	0.2	0.2	0.7	0.2	0.2	0.1	0.1	0.2
																				
dcm6/7		1	1	0	1	0	0	1	0	1	0	0	1	0	0	0	0	1	0	0
	G**C**GC	0.9	0.9	0.2	0.9	0.2	0.2	0.9	0.2	0.9	0.2	0.2	0.9	0.2	0.2	0.2	0.2	0.9	0.2	0.2
	GCG**C**	0.6	0.6	0.1	0.5	0.1	0.1	0.5	0.1	0.5	0.1	0.1	0.6	0.1	0.2	0.1	0.1	0.6	0.1	0.1
																				
dam?	CT**A**T	0.3	0.3	0.3	0.3	0.2	0.3	0.3	0.3	0.3	0.6	0.7	0.6	0.7	0.7	0.6	0.6	0.9	0.7	0.6
																				
	**C**TTC	0.1	0.1	0.1	0.1	0.1	0.1	0.1	0.1	0.1	0.3	0.3	0.3	0.3	0.3	0.3	0.3	0.6	0.3	0.2
	CTT**C**	0.2	0.2	0.2	0.2	0.1	0.2	0.2	0.2	0.2	0.3	0.3	0.3	0.3	0.3	0.3	0.3	0.6	0.3	0.3
																				
	GG**A**G	0.4	0.3	0.3	0.3	0.3	0.3	0.3	0.3	0.3	0.4	0.4	0.4	0.4	0.5	0.4	0.4	0.5	0.3	0.4
																				
	G**A**GTC	0.4	0.4	0.4	0.4	0.3	0.3	0.3	0.4	0.3	0.4	0.4	0.4	0.4	0.4	0.4	0.4	0.5	0.4	0.4
	G**A**CTC	0.4	0.3	0.3	0.3	0.3	0.3	0.3	0.3	0.3	0.5	0.5	0.5	0.6	0.5	0.5	0.5	0.3	0.4	0.5
																				
	GG**A**CC	0.5	0.5	0.5	0.5	0.4	0.4	0.5	0.5	0.5	0.4	0.4	0.4	0.4	0.4	0.4	0.3	0.3	0.3	0.5
	GGAC**C**	0.4	0.4	0.3	0.4	0.3	0.4	0.3	0.4	0.3	0.4	0.4	0.4	0.4	0.4	0.4	0.4	0.5	0.3	0.4
	GGGC**C**	0.3	0.3	0.2	0.2	0.2	0.2	0.2	0.2	0.2	0.3	0.3	0.4	0.3	0.4	0.4	0.3	0.6	0.3	0.3
	GGC**C**C	0.3	0.2	0.3	0.2	0.3	0.3	0.3	0.3	0.3	0.4	0.4	0.4	0.5	0.4	0.5	0.3	0.4	0.3	0.5
																				
dcm5		1	1	1	1	1	1	1	1	1	1	1	1	1	1	1	0	0.5	1	1
																				
dcm8		0	0	0	1	0	0	0	0	0	0	0	1	1	1	0	0	0	1	0
dam3 ***		0	0	0	1	0	0	0	0	0	0	0	1	1	1	0	0	0	1	0
																				
* putatively methylated amino acid in red; ** number of MTase gene/genome; 0.5 = truncated gene; *** dam3 also with truncations.													Meth.-Score > 0.5							

## Data Availability

Genome sequences of PG21 (acc.-no. FP236530.1), A136 (CP055143.1), FBG (CP055151.1), SP10291 (CP055149.1), SP2565 (CP055144.1), SP3615 (CP055150.1), SS10 (CP055146.1), SS25 (CP055147.1), VO31120 (CP055148.1), 475 (CP055145.1), 8958 (CP055142.1) were downloaded from NCBI (https://www.ncbi.nlm.nih.gov/nuccore/); all other draft assemblies are available at OSF (DOI: 10.17605/OSF.IO/CZRBT). All raw sequencing data and high-quality assemblies are made available under BioProject PRJNA429440.

## Supplementary Materials

The following supporting information can be downloaded at: https://www.mdpi.com/article/10.3390/microorganisms11061591/s1, Supplementary Excel File of Tables S1–S7; Supplementary Microsoft Word File of Figures S1–S6.
