# Supplementary material for: Characterisation of Type II DNA Methyltransferases of Metamycoplasma hominis
Source: Microorganisms. 2023 Jun 15;11(6):1591. doi: 10.3390/microorganisms11061591 (PMC10305163; doi:10.3390/microorganisms11061591)
Supplement: Supplementary file 1 [file microorganisms-11-01591-s001.zip › Supplementary Figures S1 to S6_R1.pdf]

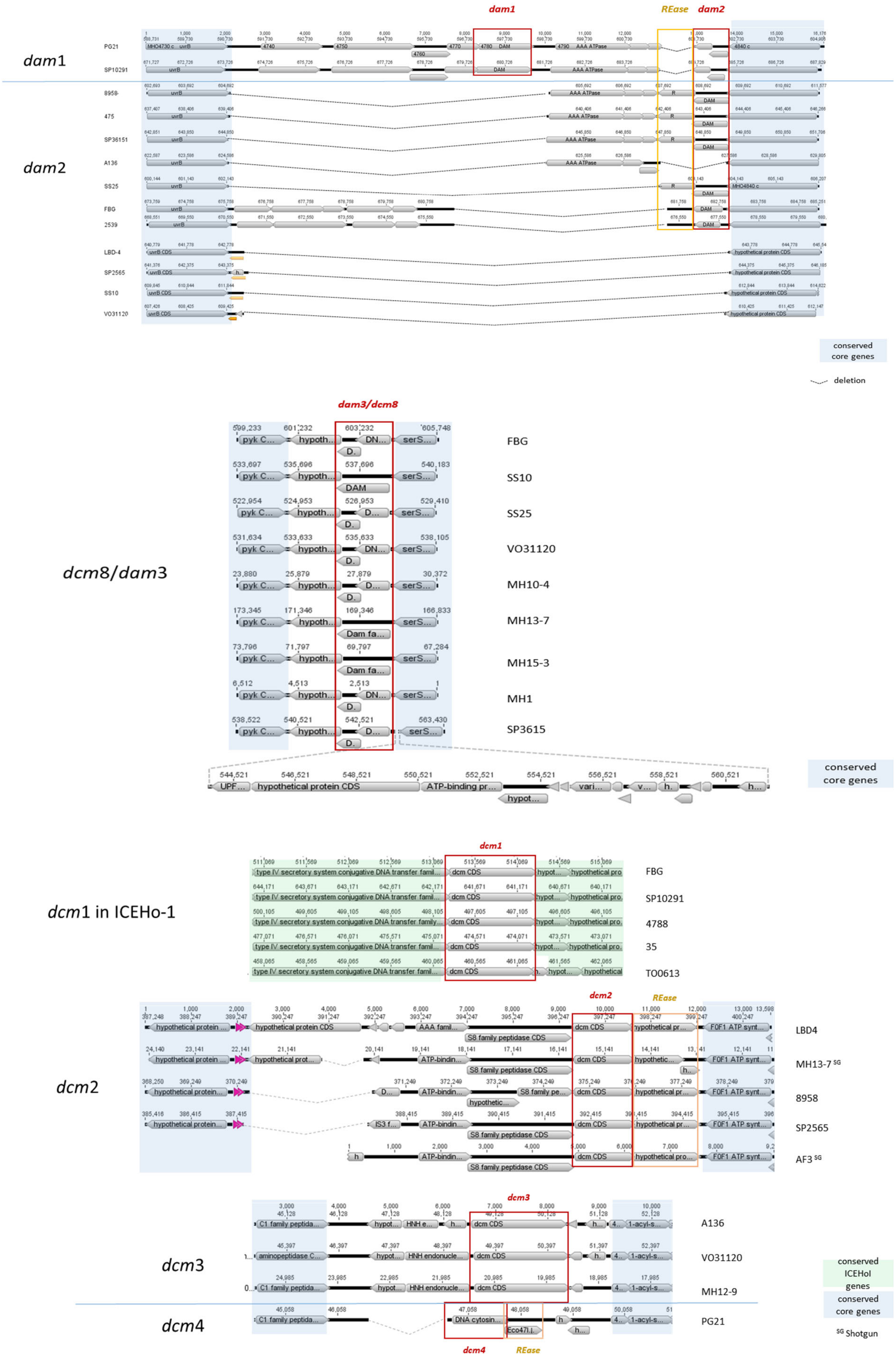
dam1
REase
dam2
dam1
PG21
SP10291
dam2
8958
475
SP36151
A136
SS25
FBG
2539
LBD-4
SP2565
SS10
VO31120
conserved core genes
deletion
dam3/dcm8
dcm8/dam3
FBG
SS10
SS25
VO31120
MH10-4
MH13-7
MH15-3
MH1
SP3615
conserved core genes
dcm1
dcm1 in ICEHo-1
FBG
SP10291
4788
35
TO0613
dcm2
REase
dcm2
LBD4
MH13-7 SG
8958
SP2565
AF3 SG
dcm3
dcm3
A136
VO31120
MH12-9
dcm4
PG21
dcm4
REase
conserved ICEHoI genes
conserved core genes
SG Shotgun

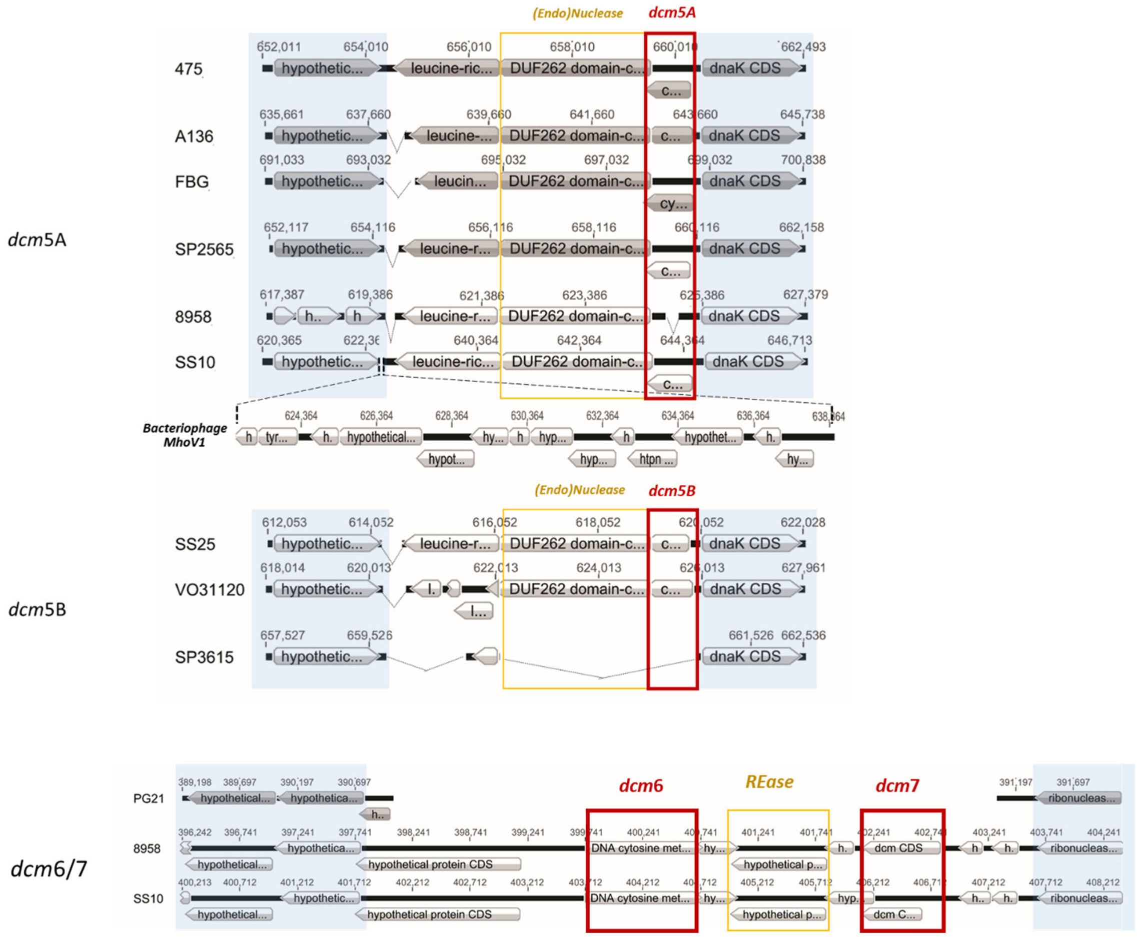

Figure S1. Gene composition in gene loci of type II DNA-MTases

Genes of the different MTase gene loci (*dam*1, *dam*2, *dcm*8/*dam*3 and *dcm*1 – *dcm6*/7) of the deep sequenced *M. hominis* genomes [1] were presented using Genious. Chromosomal core genes according to type strain PG21 are shaded in blue, ICEHo-I core genes in green, MTase gene regions framed in red and REase gene regions in yellow.

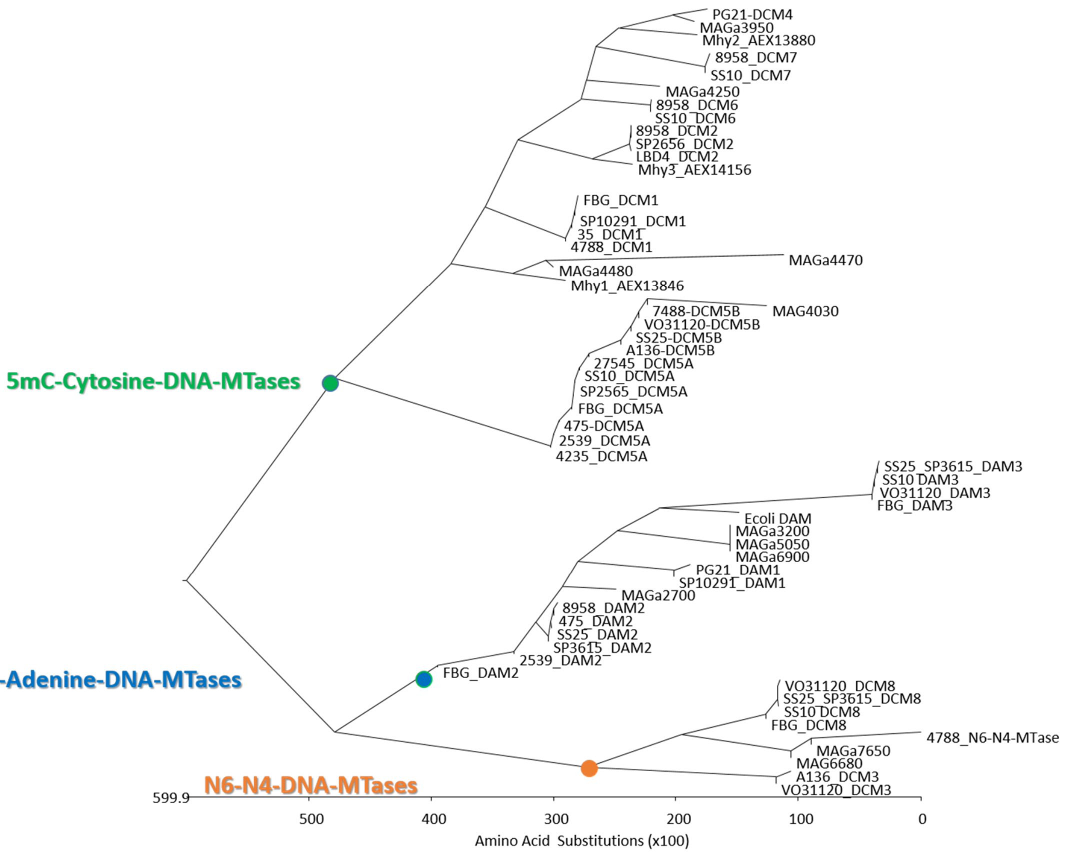

Figure S2. Phylogenetic tree of type II DNA MTases of *M. hominis*, *M. agalactiae* and *M.hyorhinis*
In addition to the postulated *M. hominis* MTases already shown in Figure 1, BlastP-derived proteins sequences of other strains of *M. hominis*, *M. agalactiae* [2] and *M. hyorhinis* [3] were aligned by ClustalW for phylogenetic tree construction.

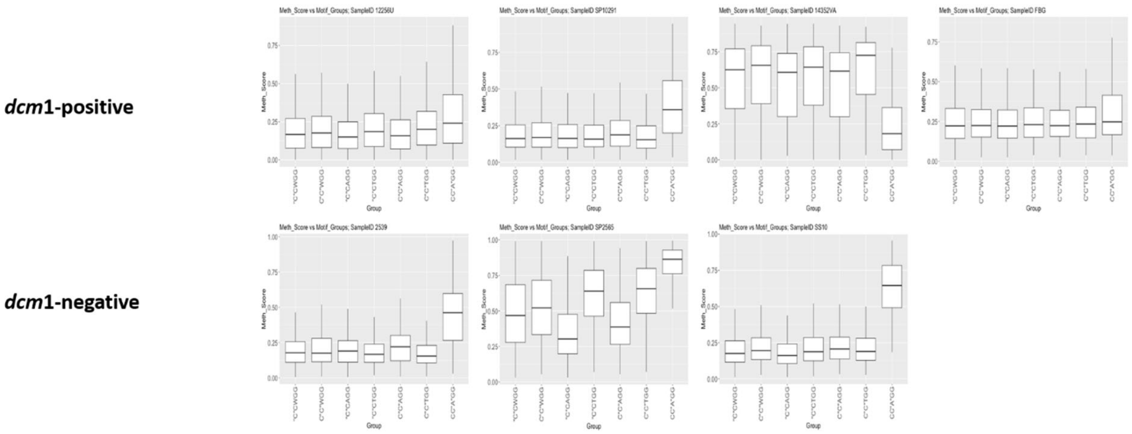

Figure S3. Tombo-calculated methylation scores of CCWGG motif variants
Methylation scores of the motifs were calculated for selected *M. hominis* isolates carrying the *dcm*1-gene (*dcm*1-positive) or without *dcm*1-gene (*dcm*1-negative). Position of the calculated 5mC or 6mA nucleotide is marked by “C” or “A”.

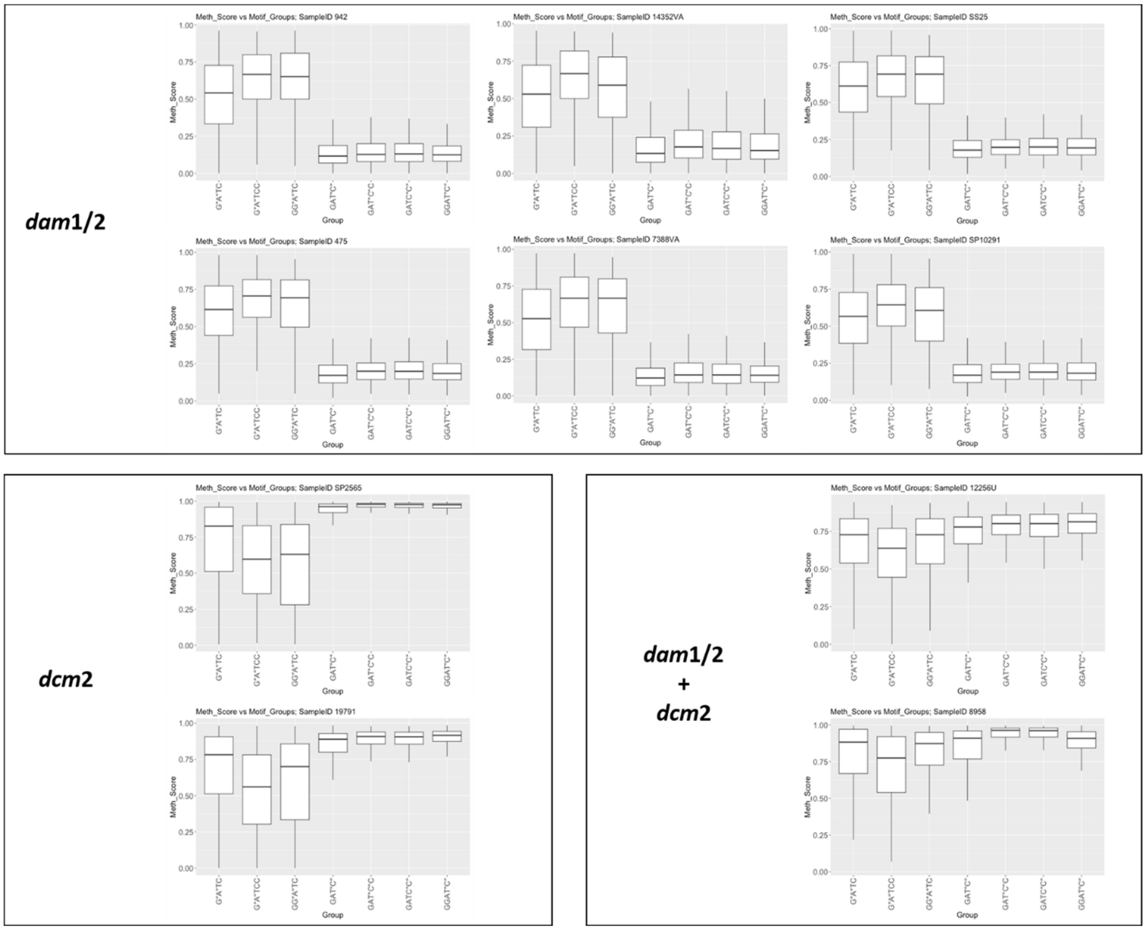

Figure S4. Tombo-calculated methylation scores of GATC(C) motif variants

Methylation scores of the motifs were calculated for selected *M. hominis* isolates carrying *dam*1 or *dam*2 (***dam*1/2**) and *dcm*2 genes without dam1/2 (***dcm*2**) or with *dam*1/2 (***dam*1/2 + *dcm*2**). Position of the calculated 5mC or 6mA nucleotide is marked by "C" or "A".

A.

MTase	MTase-Motif	Restriction enzyme[a]			Number of MTase gene copies / isolate's genome[b]			
		Cut1: °X	Cut2: ^{m}X	Cut3: $^{+}$X	**12256U**	**4518**	**13856**	**8958**
DCM2	GAT^{m}C	*Dpn*II G^{+}AT°C	-	*Sau*3AI G°AT^{+}C	1	0	1	1
DAM1	G^{m}ATC	*Sau*3AI G°AT^{+}C	*Dpn*I G^{m}AT^{+}C	*Mbo*I G^{+}AT°C	1	1	0	0
DAM2					F	0	1	1

[a] Restriction: independent on methylation of AA X (°X), requires methylation of AA X (^{m}X) or is inhibited by methylation of AA X ($^{+}$X); [b] F = fragmented gene, T = truncated gene

B.

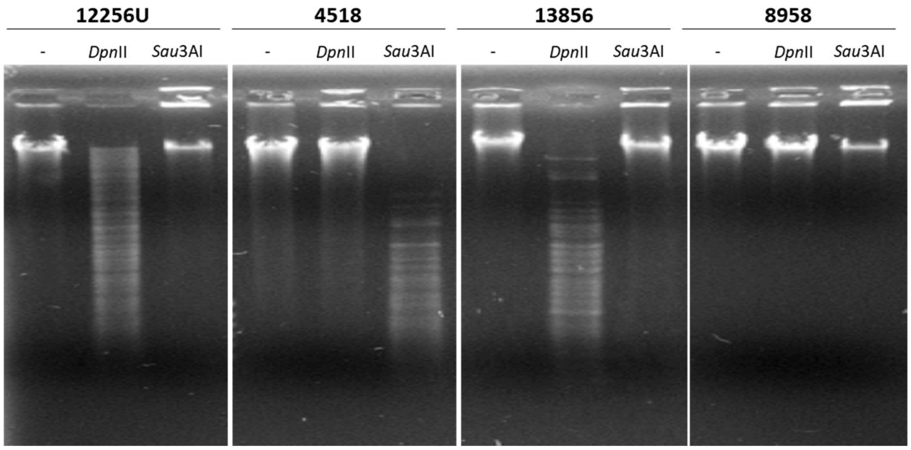

Figure S5. Methylation sensitive restriction for proof of DCM2 GAT^{m}C-activity

M. hominis strains 12256U, 4518, 13856 and 8958 were subjected to methylation sentsitive restriction. (A) Table of DCM2, DAM1 and DAM2 gene presence and methylation specificities of restriction enzymes. (B) 1% (w/v) agarose gel of the genomic DNA: unrestricted DNA (-); restricted with *Dpn*II, which cuts independent on 5mC methylation, *Sau*3AI, which is inhibited by 5mC methylation of GATC. 4518 serves as a control, as its DNA is digested by *Sau*3AI due to absence of DCM2. DNA of strain 8958 is not cut by *Dpn*II due to his DAM2 activity; whereas DNA of strains 12256U and 13856 is cut by *Dpn*II indicating absence of 6mA methylation in the GATC motif and respectively, absence of DAM1 or DAM2 methylation activity.

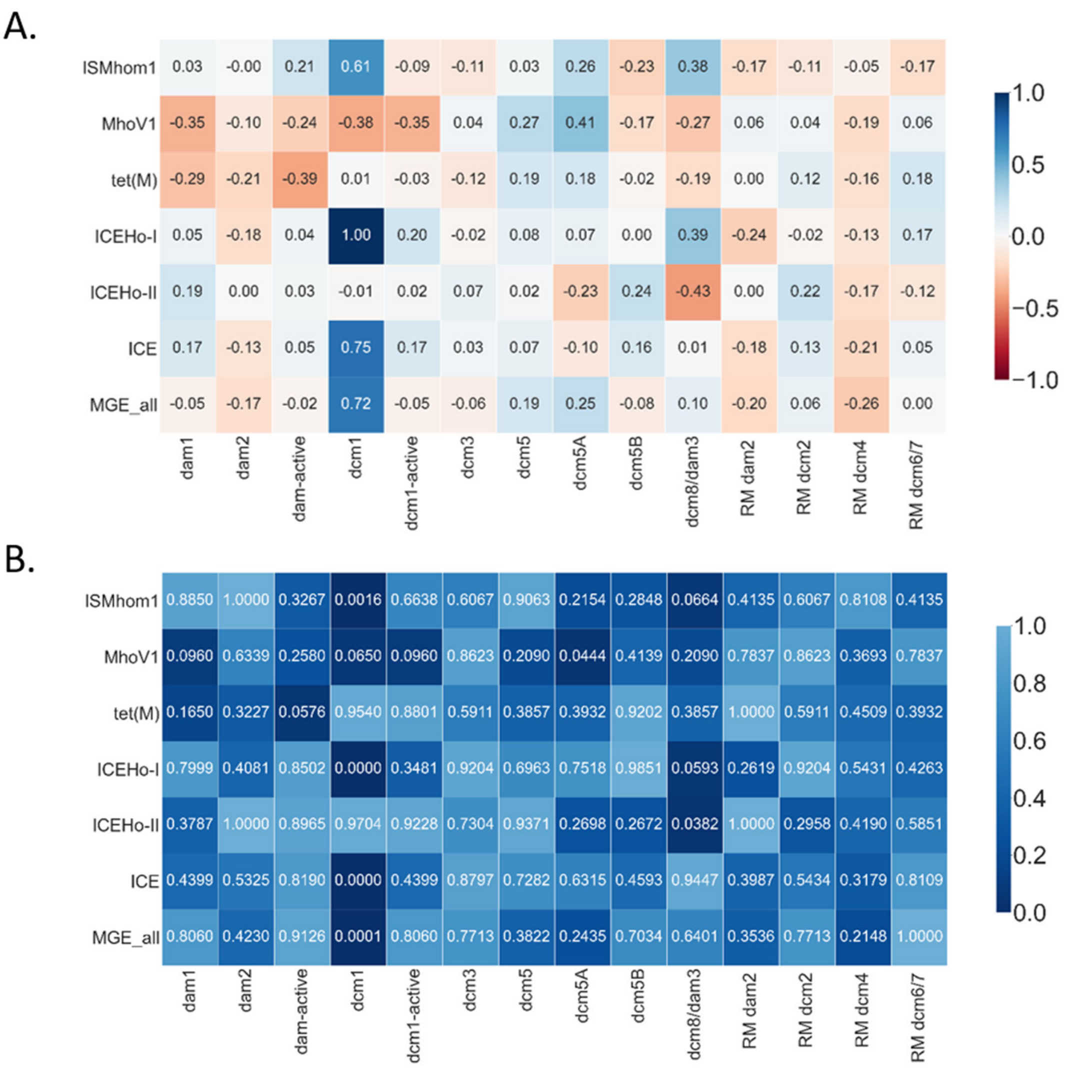

Figure S6. Correlation matrices of MTases and MGEs

Presence of MTases was correlated with number of MGEs in 23 *M. hominis* isolates and type strain PG21. Pearsons correlations coefficient (A) and p-values (B) are shown for each analysed correlation. Raw data are shown in Supplementary Table S6.

1. Henrich, B.; Hammerlage, S.; Scharf, S.; Haberhausen, D.; Fürnkranz, U.; Köhrer, K.; Peitzmann, L.; Fiori, P.L.; Spergser, J.; Pfeffer, K.*, et al.* Characterisation of mobile genetic elements in mycoplasma hominis with the description of iceho-ii, a variant mycoplasma integrative and conjugative element. *Mobile DNA* **2020**, *11*, 30.
2. Dordet-Frisoni, E.; Vandecasteele, C.; Contarin, R.; Sagné, E.; Baranowski, E.; Klopp, C.; Nouvel, L.-X.; Citti, C. Impacts of mycoplasma agalactiae restriction-modification systems on pan-epigenome dynamics and genome plasticity. *Microbial Genomics* **2022**, *8*.
3. Chernov, A.V.; Reyes, L.; Xu, Z.; Gonzalez, B.; Golovko, G.; Peterson, S.; Perucho, M.; Fofanov, Y.; Strongin, A.Y. Mycoplasma cg- and gatc-specific DNA methyltransferases selectively and efficiently methylate the host genome and alter the epigenetic landscape in human cells. *Epigenetics* **2015**, *10*, 303-318.
